# Branching and converging pathways in fungal natural product biosynthesis

**DOI:** 10.1186/s40694-022-00135-w

**Published:** 2022-03-07

**Authors:** Xingxing Wei, Wei-Guang Wang, Yudai Matsuda

**Affiliations:** 1 Department of Chemistry, City University of Hong Kong, Tat Chee Avenue, Kowloon, Hong Kong SAR China; 2 Key Laboratory of Chemistry in Ethnic Medicinal Resources, State Ethnic Affairs Commission and Ministry of Education and Key Laboratory of Natural Products Synthetic Biology of Ethnic Medicinal Endophytes, State Ethnic Affairs Commission, Yunnan Minzu University, Kunming, 650031 China

**Keywords:** Natural products, Biosynthesis, Branching and converging pathways

## Abstract

In nature, organic molecules with great structural diversity and complexity are synthesized by utilizing a relatively small number of starting materials. A synthetic strategy adopted by nature is pathway branching, in which a common biosynthetic intermediate is transformed into different end products. A natural product can also be synthesized by the fusion of two or more precursors generated from separate metabolic pathways. This review article summarizes several representative branching and converging pathways in fungal natural product biosynthesis to illuminate how fungi are capable of synthesizing a diverse array of natural products.

## Introduction

Structural diversity is a characteristic feature of naturally occurring organic compounds (natural products). Natural products possess a wide range of biological activities that can be attributed to their diverse and complicated molecular architectures. In nature, a great array of natural products are synthesized from a relatively small number of starting materials derived from primary metabolism. One reason behind the structural diversity of natural products is the presence of a significant number of core synth(et)ases, which are involved in the backbone synthesis of natural products. Core synth(et)ases include polyketide synthases (PKSs), nonribosomal peptide synthetases (NRPSs), and terpene cyclases and are often responsible for the first committed step of a natural product biosynthesis, providing branching points between primary and secondary metabolic pathways. For example, PKSs accept limited starter and extender units, as represented by acetyl-coenzyme A (CoA) and malonyl-CoA, and perform repeated condensation of C_2_ units to generate polyketide products with diverse chain lengths, oxidation levels, or cyclization patterns [1, 2]. Although polyketide biosynthesis resembles that of fatty acids, an incomparable number of molecules can be produced by PKSs [3]. Likewise, terpene cyclases can cyclize achiral and linear substrates, such as polyprenyl pyrophosphates and (oxido)squalene, in a diverse manner, generating great structural diversity in terpene natural products [4].

**Fig. 1:**
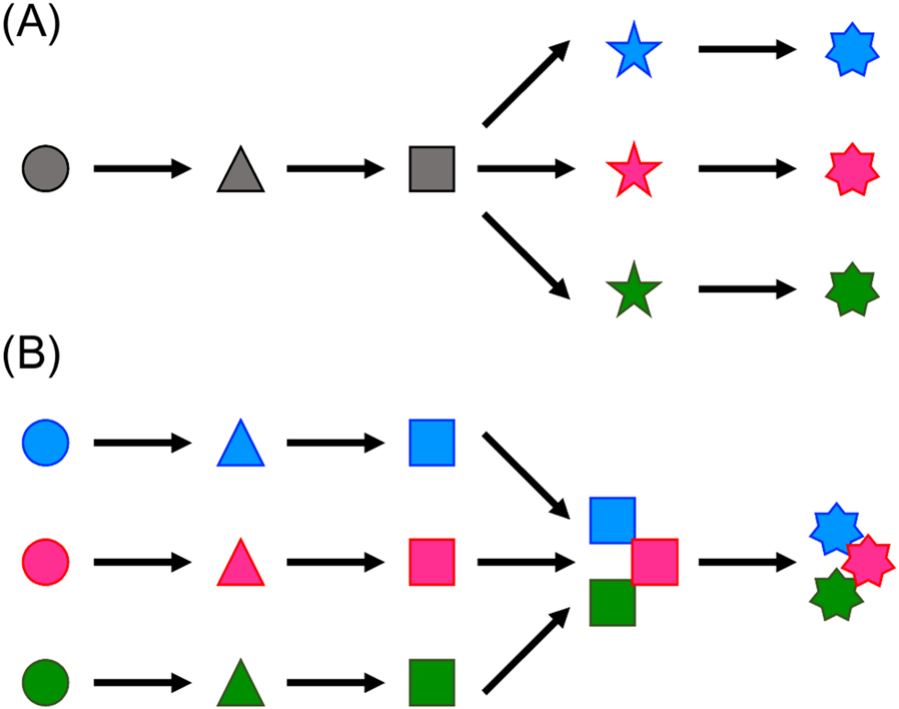
Schematic illustration of **A** branching and **B** converging pathways

In addition to structural diversification by core synth(et)ases, nature also uses other synthetic strategies to afford a variety of natural products [5]. Pathway branching is one such example, in which a molecule is synthesized as a common biosynthetic precursor and then undergoes distinct tailoring reactions to generate several different natural products (Fig. 1A). In another biosynthetic process, two or more molecules synthesized in separate pathways are combined together to provide a single natural product (Fig. 1B). In other words, convergent biosynthesis is nature’s alternate approach to expand natural product diversity. Recent advances in genome sequencing technology and the development of molecular biological tools have allowed the elucidation of biosynthetic pathways and the synthesis of many natural products, including those synthesized in branching and converging pathways. In this review article, we summarize representative examples of branching and converging pathways in fungal natural product biosynthesis, with the aim to highlight nature’s sophisticated strategies for synthesizing diverse molecules.

## Branching pathways

As mentioned above, core synth(et)ases utilize substrates also used by the enzymes involved in primary metabolism but synthesize diverse molecules, thus greatly contributing to pathway divergence in natural product biosynthesis. As the functions and diversity of core synth(et)ases are well summarized elsewhere [1, 2, 4], this review focuses on the biosynthetic processes in which pathway branching occurs at the mid- or late-biosynthesis stage, especially those in which a single molecule serves as a common precursor of more than two different pathways.

### Fungal xanthones

**Fig. 2:**
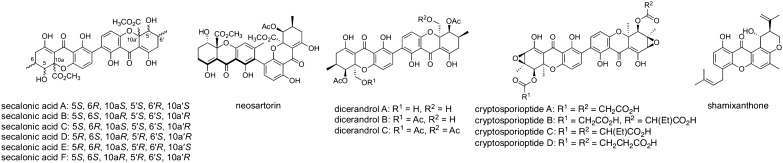
Representative fungal xanthones

Xanthone is a tricyclic organic molecule, and xanthone derivatives are a class of natural products that exhibit a wide range of biological activities such as anti-bacterial activities and cytotoxicity [6, 7]. Fungi are prolific producers of natural products with a xanthone (including di- and tetrahydroxanthone [THX]) scaffold (Fig. 2). In recent years, biosynthetic gene clusters of several fungal xanthones have been identified and characterized, revealing how fungi produce diverse xanthone compounds [8–16]. Although the xanthone skeleton can be synthesized through several different pathways in nature, all fungal xanthones are considered to be derived from the anthraquinone chrysophanol (**1**), which is generated by seven enzymes from one acetyl-CoA molecule and seven malonyl-CoA molecules [12].

**Fig. 3:**
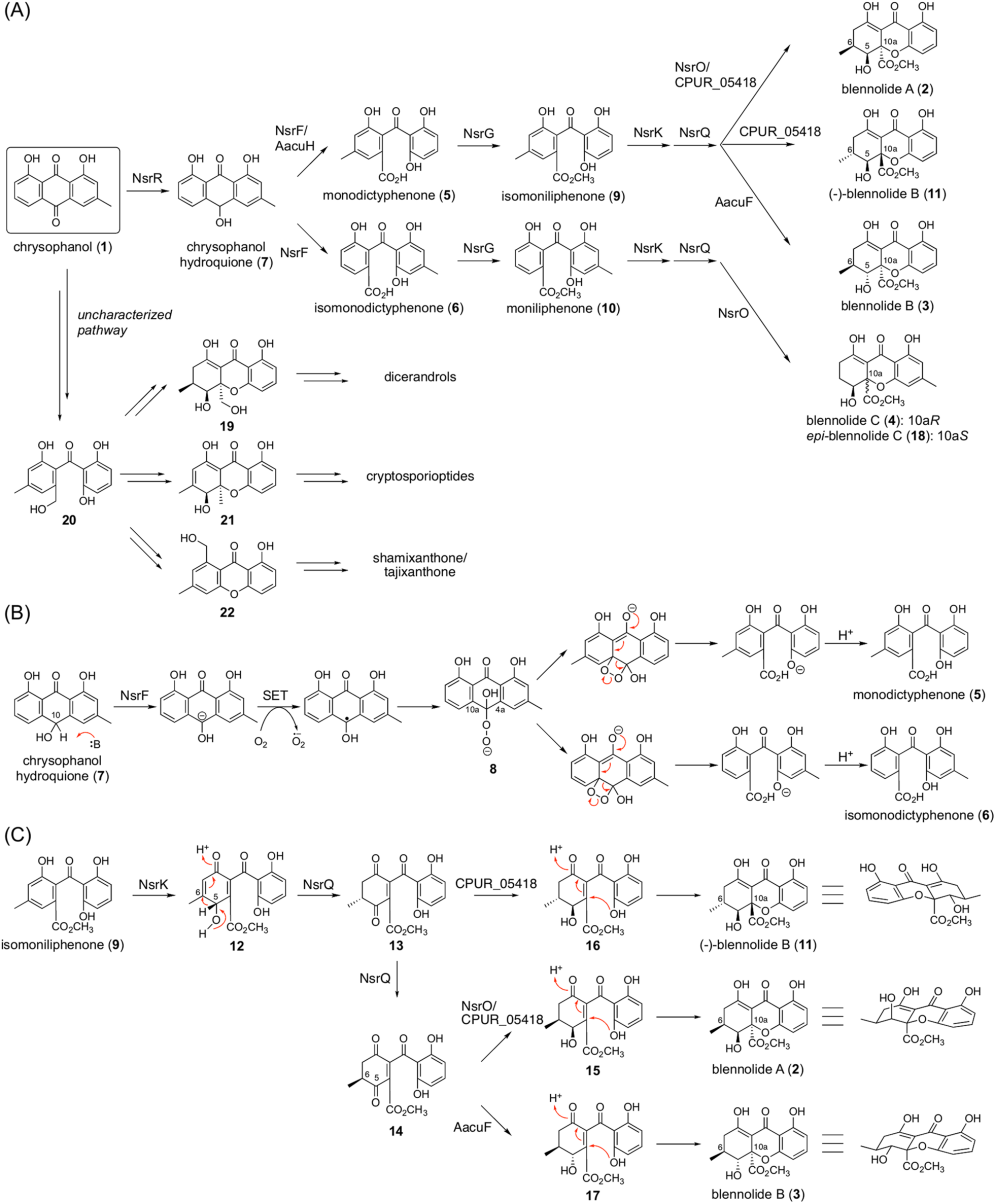
Biosynthesis of chrysophanol-derived fungal xanthones. **A** Biosynthetic pathways of diverse fungal xanthones. **B** Proposed reaction mechanism of NsrF. **C** Biosynthetic mechanisms to provide blennolides

Blennolides A-C (**2**–**4**) are representative fungal THXs and the precursors of many THX dimers [17], such as secalonic acids and neosartorin [18–20]. In the biosynthesis of neosartorin, chrysophanol (**1**) is transformed into monodictyphenone (**5**) and isomonodictyphenone (**6**), providing a branching point (Fig. 3A) [12]. Previously, it was thought that NsrF serves as a Baeyer–Villiger monooxygenase to insert an oxygen atom at two different positions in **1** [12, 15]. However, the recent in-depth characterization of GedF, an NsrF homologue in geodin biosynthesis, indicated that chrysophanol is not a direct NsrF substrate [21]. It is currently proposed that **1** is first reduced to chrysophanol hydroquinone (**7**) by the short-chain dehydrogenase/reductase (SDR) NsrR, which is also required for chrysophanol formation. NsrF then accepts **7** to deprotonate from the C-10 position, followed by single electron transfer (SET) from the carbanion to molecular oxygen to generate the peroxy anion **8**. The peroxy anion **8** subsequently attacks either the C-4a or C-10a position, followed by bond migration to yield **5** or **6**, respectively (Fig. 3B). Interestingly, AacuH, the NsrF homologue engaged in secalonic acid biosynthesis, selectively generates **5** [16], indicating that this enzyme controls Michael addition by the peroxy anion.

Monodictyphenone (**5**) and isomonodictyphenone (**6**) undergo methyl-esterification to afford 2,2′,6′-trihydroxy-4-methyl-6-methoxyacyldiphenylmethanone (**9**; hereby termed isomoniliphenone) and moniliphenone (**10**), respectively, both of which are key blennolide precursors (Fig. 3A). Compound **9** can be converted into blennolide A (**2**), blennolide B (**3**), and ( −)-blennolide B (**11**) (Fig. 3C) [15]. In the neosartorin pathway, **9** is almost exclusively transformed to **2**, in which three enzymes, the flavin-dependent monooxygenase (FMO) NsrK, the isomerase NsrQ, and the SDR NsrO, collaboratively synthesize the THX scaffold [22]. In this biosynthetic process, NsrK initially performs hydroxylation at the C-5 position, and the resultant hydroxycyclohexadienone **12** undergoes NsrQ-catalyzed isomerization to provide the cyclohexanedione intermediate **13**. NsrQ subsequently inverts the stereochemistry at the C-6 position to give **14**. NsrO then reduces the C-5 carbonyl of **14** to the (5*S*)-hydroxy group to yield **15**, followed by spontaneous heterocyclization to yield **2**. This cyclization appears to occur in a stereoselective manner to avoid the 1,3-axial–pseudoaxial interaction between the C-6 methyl group and the C-10a methyl ester. Intriguingly, CPUR_05418, a NsrO homologue in *Claviceps purpurea*, accepts the first isomerized product of NsrQ, **13**, and serves as the C-5*S* reductase to generate **11** with a (5*S*,6*R*,10a*S*) configuration via **16**. Furthermore, the *Aspergillus aculeatus* secalonic acid pathway adopts two SDRs with distinct stereoselectivity [14]. One SDR, AacuD, is homologous to NsrO and responsible for blennolide A formation, whereas the other SDR AacuF conducts the C-5*R* reduction, leading to the production of **3** possessing the (5*R*,6*S*,10a*R*) configuration via **17** [16]. In addition, **10** undergoes similar reactions catalyzed by NsrK, NsrQ, and NsrO to yield blennolide C (**4**) and its C-10a epimer *epi*-blennolide C (**18**) [15]; cyclization occurs in a nonselective manner, probably due to lack of the C-6 methyl group.

Chrysophanol (**1**) appears to be the precursor of many other fungal xanthones whose biosynthetic pathways have not been fully elucidated. On the basis of the dicerandrol structure [23, 24], their biosynthesis should involve a precursor that is analogous to blennolide A (**2**) but contains a hydroxymethyl group instead of a methyl ester, **19** (Fig. 3A), which is derived from a monodictyphenone analogue **20** with a hydroxymethyl group. Compound **20** could also be utilized as a common biosynthetic intermediate for other fungal xanthones, such as cryptosporioptide (via **21**) [14] and tajixanthone/shamixanthone (via **22**) [8], although the mechanism to provide the hydroxymethyl group has yet to be elucidated.

### Ergot alkaloids

**Fig. 4:**
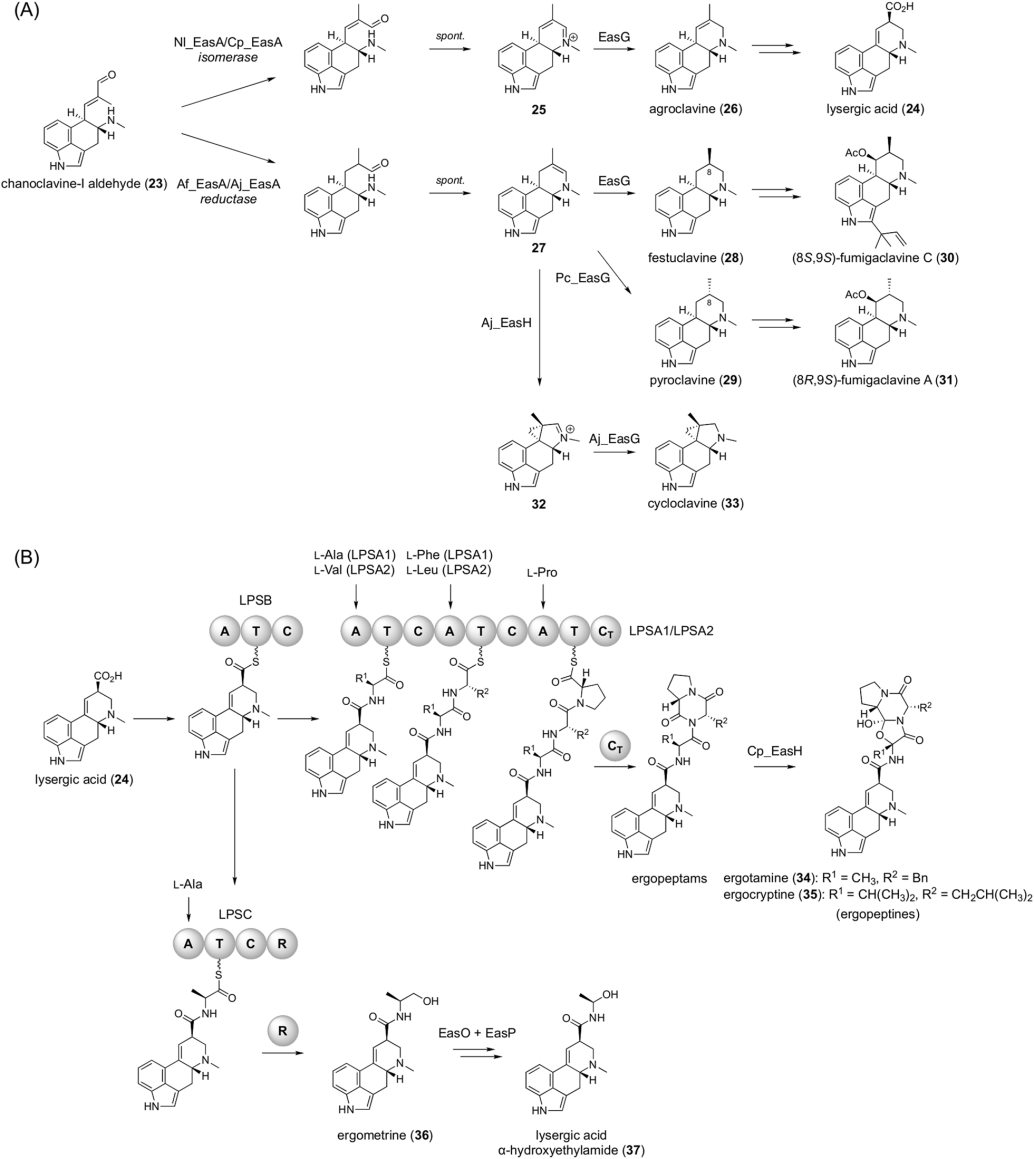
Biosynthesis of ergot alkaloids. **A** Branched biosynthesis of ergot alkaloids in filamentous fungi. **B** Biosynthesis of ergopeptines and lysergic acid amides in *Claviceps*

Ergot alkaloids are a diverse group of fungal indole alkaloids with pharmaceutical and agricultural value [25, 26]. They are synthesized by filamentous fungi of several different genera and provide another example in which pathway branching significantly contributes to structural diversification. All ergot alkaloids are believed to be biosynthesized from chanoclavine-I aldehyde (**23**) as a common precursor (Fig. 4A), which is generated by five dedicated enzymes using l-tryptophan and dimethylallyl pyrophosphate (DMAPP) as starting materials.

Chanoclavine-I aldehyde (**23**) is converted into lysergic acid (**24**) in several fungi, such as *Epichloë lolii* and *Claviceps purpurea*. In this transformation, **23** is accepted by EasA (Nl_EasA or Cp_EasA) to undergo double-bond isomerization, followed by spontaneous cyclization [27, 28]. The resultant iminium cation **25** is then reduced by the SDR EasG to provide agroclavine (**26**) [29], which is further converted to **24** (Fig. 4A). Meanwhile, in *Aspergillus* or *Penicillium* species, the ergot alkaloid pathways use an EasA homologue (Af_EasA or Aj_EasA) with reductase activity to provide **27** (Fig. 4A) [27, 28, 30, 31]. The catalytic tyrosine residue in the reductase version of EasA is substituted with phenylalanine in the isomerase-type EasA [28], which cannot perform the reduction reaction, thus causing functional differences in the EasA homologues. In *Aspergillus fumigatus*, **27** is predominantly converted to festuclavine (**28**) by EasG (Af_EasG), whereas a considerable amount of pyroclavine (**29**), the C-8 epimer of **28**, is also produced by the homologous enzyme Pc_EasG in *Penicillium commune* [32]. Compounds **28** and **29** undergo further tailoring reactions to give (8*S*,9*S*)-fumigaclavine C (**30**) in *A. fumigatus* and (8*R*,9*S*)-fumigaclavine A (**31**) in *P. commune*, respectively, as the end products. Intriguingly, in *A. japonicus*, another enzyme, EasH (Aj_EasH), which is an α-ketoglutarate (αKG)-dependent dioxygenase, acts before the EasG reduction to catalyze structural rearrangement to install the cyclopropane moiety to yield **32**, eventually leading to the production of cycloclavine (**33**) [31].

Lysergic acid (**24**) undergoes further modification by several NRPSs to yield ergopeptines and lysergic acid amides (Fig. 4B). In *C. purpurea*, **24** is activated by and loaded onto a single-module NRPS, LPSB, with adenylation (A)-thiolation (T)-condensation (C) domain organization [33–35]. The activated **24** is then transferred to the assembly line of LPSA1 or LPSA2, two other NRPSs homologous to each other [35]. After the incorporation of three amino acids, the elongated peptide chain is released by the terminal condensation-like (C_T_) domain to furnish the diketopiperazine structure. The resultant ergopeptams are then transformed into ergopeptines, namely ergotamine (**34**) and ergocryptine (**35**), by the αKG-dependent dioxygenase Cp_EasH [36]. LPSA1 and LPSA2 have the same domain architecture but utilize different combinations of amino acids. It has been proposed that the three LPSA1 A domains activate l-alanine, l-phenylalanine, and l-proline, and that those of LPSA2 recognize l-valine, l-leucine, and l-proline [37]. Furthermore, LPSB-loaded **24** can be utilized by another single module NRPS with A-T-C-reductase (R) domain organization, LPSC, thus incorporating l-alanine and forming ergometrine (**36**) by reductive release of the peptide chain [34]. In some fungi, such as *Claviceps paspali*, **36** can be further transformed into lysergic acid α-hydroxyethylamide (**37**; LAH), which is thought to be produced by the FMO EasO and α/β hydrolase-fold enzyme EasP [38].

### Trichothecenes

**Fig. 5:**
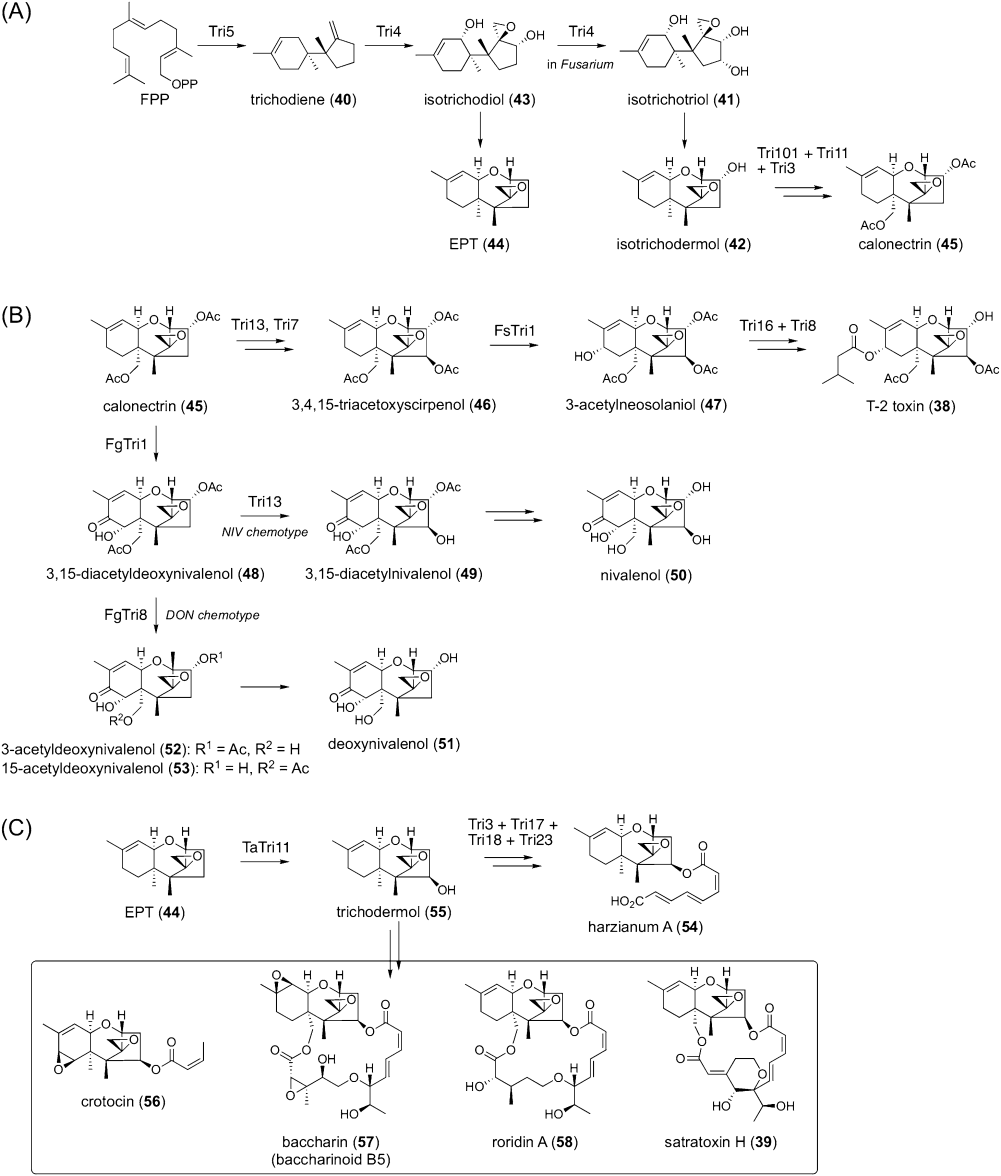
Biosynthesis of trichothecenes. **A** Biosynthesis of EPT (**44**) and calonectrin (**45**). **B** Biosynthesis of the T-2 toxin (**38**), nivalenol (**50**), and deoxynivalenol (**51**) in *Fusarium*. **C** Biosynthesis of harzianum A (**54**) and related natural products

Trichothecenes are a large group of sesquiterpenoid mycotoxins produced by a variety of fungi of different genera and represented by T-2 toxin (**38**) and satratoxin H (**39**) (Fig. 5) [39, 40]. The trichothecene core structure features an epoxide ring at the C-12/C-13 positions, which is crucial for their biological activities. The first committed biosynthesis step of all trichothecenes is the cyclization of farnesyl pyrophosphate (FPP) into the sesquiterpene hydrocarbon trichodiene (**40**), which is catalyzed by the terpene cyclase Tri5 (Fig. 5A) [41, 42]. The sesquiterpene **40** then undergoes multiple oxidations catalyzed by the cytochrome P450 monooxygenase Tri4. In *Fusarium* fungi, Tri4 is responsible for four successive oxidative reactions to afford isotrichotriol (**41**), which spontaneously cyclizes to isotrichodermol (**42**) [43]. Contrarily, Tri4 in other fungi only performs three rounds of oxidations to yield isotrichodiol (**43**), which is nonenzymatically transformed into 12,13-epoxytrichothec-9-ene (**44**; EPT) [43, 44]. Thus, functional differences in Tri4 provide the first branching point in fungal trichothecene biosynthesis.

Isotrichodermol (**42**) in *Fusarium* is subsequently converted to calonectrin (**45**) by three enzymes, the acetyltransferase Tri101 [45], the P450 Tri11 [46], and the acetyltransferase Tri3 [47, 48] (Fig. 5A). In *Fusarium sporotrichioides*, a known producer of the T-2 toxin (**38**), **45** is converted to 3,4,15-triacetoxyscirpenol (**46**) by P450 Tri13 [49] and the acetyltransferase Tri7 [50] (Fig. 5B). Then, **46** is hydroxylated by P450 Tri1 (FsTri1) to give 3-acetylneosolaniol (**47**) [51]. Finally, the acyltransferase Tri16 [52], which installs the isovaleryl side-chain that possibly utilizes isovaleryl-CoA, and the esterase Tri8 complete the biosynthesis to afford the **38** [53]. Meanwhile, another *Fusarium* species, *F. graminearum*, also employs **45** as a key biosynthetic intermediate for trichothecenes but produces different molecules than *F. sporotrichioides* (Fig. 5B). *F. graminearum* is further classified into two major types based on their metabolic profiles: the deoxynivalenol (DON) and nivalenol (NIV) chemotypes [54]. In both chemotypes, **45** first undergoes multiple oxidations to be converted to 3,15-diacetyldeoxynivalenol (**48**). This transformation is catalyzed by P450 Tri1 (FgTri1) [55], which has a function distinct from its corresponding enzyme (FsTri1) in the T-2 toxin pathway. The difference between the DON and NIV chemotypes is attributed to P450 Tri13 inactivation in the DON chemotype [49, 56]. In the NIV chemotype, Tri13 hydroxylates **48** to yield 3,15-diacetylnivalenol (**49**), which is further transformed into nivalenol (**50**). Meanwhile, in the DON chemotype, **48** undergoes deacetylation to yield deoxynivalenol (**51**) via 3-acetyldeoxynivalenol (**52**) or 15-acetyldeoxynivalenol (**53**).

In fungal species other than *Fusarium*, EPT (**44**), an isotrichodermol deoxy analogue, is used as a common precursor of trichothecenes, which often have side chain(s) derived from the polyketide pathway (Fig. 5C). In *Trichoderma arundinaceum*, which produces harzianum A (**54**), **44** is hydroxylated by P450 Tri11 (TaTri11) to give trichodermol (**55**) [57], which is further transformed into **54**. This acylation reaction requires at least four enzymes, namely PKS Tri17, P450 Tri23, and two acyltransferases, Tri3 and Tri18 [58]. Although the detailed acylation mechanism has yet to be clarified, it has been proposed that Tri17 synthesizes octa-2,4,6-trienoic acid, which is oxidized to octa-2,4,6-trienedioic acid by Tri23. An uncharacterized CoA ligase transforms dicarboxylic acid into its CoA form to be utilized by Tri3 and Tri18 to afford the acylated product **54**. Importantly, **55** also serves as the key precursor of many other acylated trichothecenes with diverse side chains, including crotocin (**56**) [59], baccharin (baccharinoid B5) (**57**) [60], roridin A (**58**) [61], and satratoxin H (**39**) [62] (Fig. 5C).

### Dioxafenestrane sesquiterpenoids

**Fig. 6:**
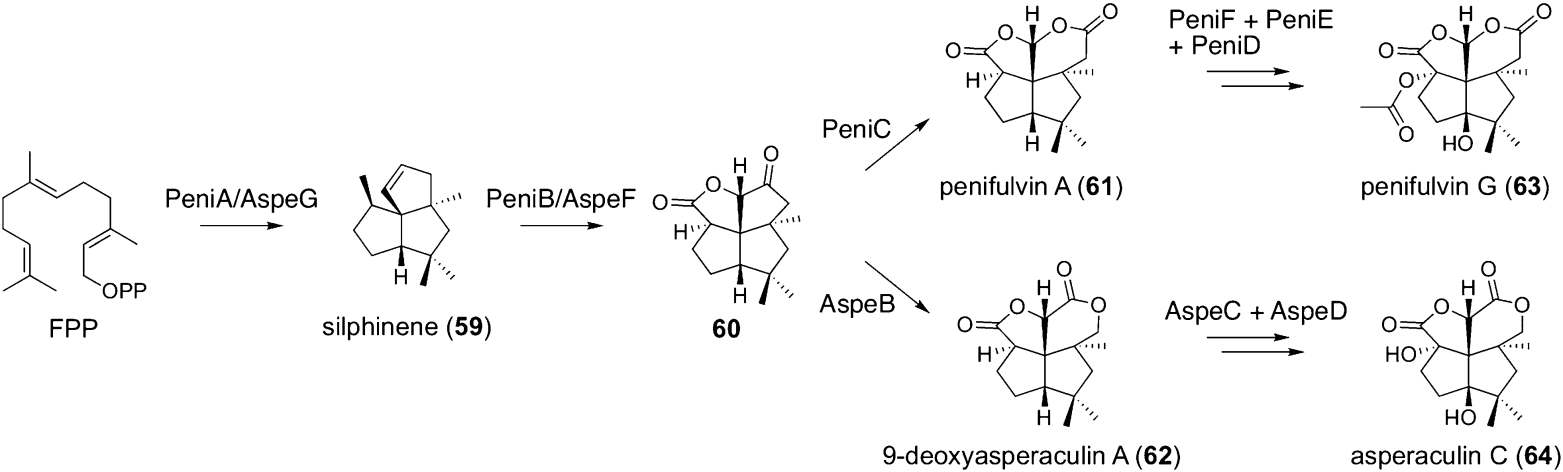
Biosynthesis of dioxafenestrane sesquiterpenoids

The dioxafenestrane sesquiterpenoid pathways provide another example in which fungal sesquiterpenoids are generated in branching pathways (Fig. 6) [63, 64]. The biosyntheses of penifulvins and asperaculins both begin with the cyclization of FPP into the sesquiterpene silphinene (**59**), which is synthesized by the terpene cyclase PeniA/AspeG. Subsequently, the P450 PeniB/AspeF performs a series of oxidative reactions to yield the tetracyclic sesquiterpenoid **60**. Compound **60** then undergoes Baeyer–Villiger oxidation in both pathways, but an oxygen atom is inserted at two distinct positions by the FMO, PeniC or AspeB, to provide penifulvin A (**61**) and 9-deoxyasperaculin A (**62**), respectively. Interestingly, although PeniC and AspeB, the key enzymes for the pathway divergence in the dioxafenestrane sesquiterpenoid biosynthesis, are both FMOs, they are phylogenetically distantly related to each other. In the late-stage of biosynthesis, **61** is converted into penifulvin G (**63**) by two αKG-dependent dioxygenases PeniD and PeniF and the acetyltransferase PeniE, whereas AspeC and AspeD, which are PeniF and PeniD homologues, respectively, oxidize **62** to form asperaculin C (**64**).

### Fusidane antibiotics

**Fig. 7:**
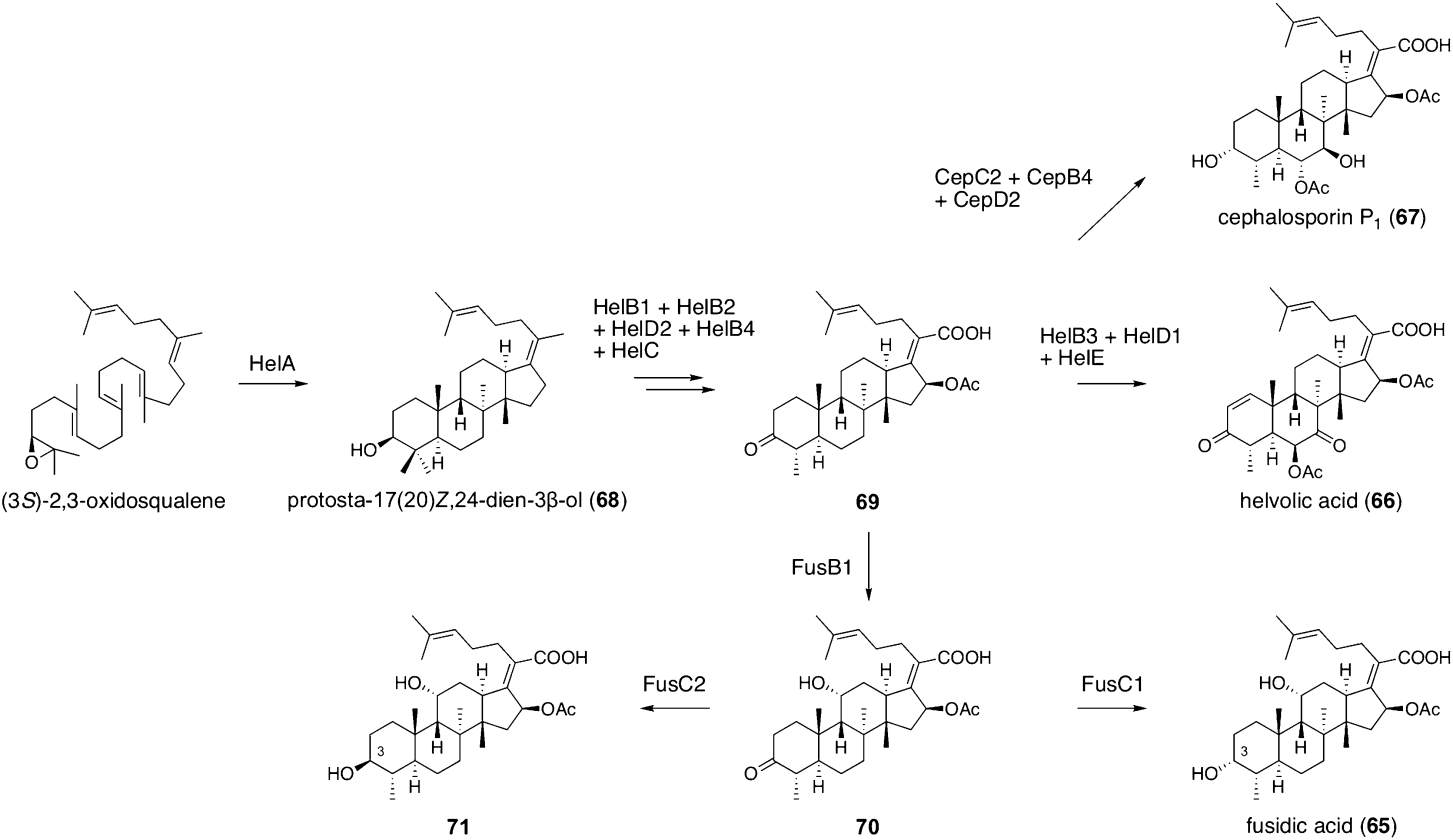
Biosynthesis of fusidane antibiotics

Fusidane antibiotics are fungal tetracyclic triterpenoids that inhibit bacterial protein biosynthesis [65], and fusidic acid (**65**), a representative fusidane antibiotic, has been utilized as an antibiotic mainly for staphylococcal infections [66]. Among fusidane antibiotics, the complete biosynthetic pathway of helvolic acid (**66**) was initially elucidated, which facilitated the biosynthesis studies of other members, such as **65** and cephalosporin P_1_ (**67**). In the fusidane antibiotic biosynthetic process (Fig. 7), (3*S*)-2,3-oxidosqualene is first cyclized by an oxidosqualene cyclase (HelA in the helvolic acid pathway) to generate the tetracyclic triterpene alcohol protosta-17(20)*Z*,24-dien-3β-ol (**68**) [67]. This triterpene alcohol undergoes several tailoring reactions to provide a common precursor for **65**, **66**, and **67** with the fusidane skeleton [68–70]; this transformation involves the three P450s, HelB1, HelB2, and HelB4, the acetyltransferase HelD2, and the SDR HelC in helvolic acid biosynthesis. The common intermediate **69** is accepted by the P450 HelB3, the acetyltransferase HelD1, and the 3-ketosteroid-∆^1^-dehydrogenase HelE to afford **66** in *Aspergillus fumigatus* [68], whereas it is converted to **67** by the SDR CepC2, the P450 CepB4, and the acetyltransferase CepD2 in *Acremonium chrysogenum* [70]. The biosynthesis of **65** in *Acremonium fusidioides* requires the P450 FusB1 and the SDR FusC1 [69]. Interestingly, another SDR FusC2, which is a HelC homologue, accepts the same substrate as FusC1, **70**, to generate **71**, the C-3 epimer of **65** with a (3*S*)-hydroxy group [69]. However, in the coexistence of FusC1 and FusC2, FusC1 works much more efficiently than FusC2, and therefore, **65** is the dominant end product in *A. fusidioides*.

### Meroterpenoids

Meroterpenoids are natural hybrid products that are in part derived from terpenoid biosynthesis and ubiquitously distributed both in primary and secondary metabolism [71–73]. Fungi are especially prolific producers of meroterpenoids, and fungal meroterpenoids exhibit enormous structural diversity, often with complicated molecular architectures and a broad range of biological activities. Fungal meroterpenoid biosynthesis occurs in a highly branching manner, and branching can be observed at different biosynthesis stages, thus allowing for the biogenesis of diverse metabolites from a small number of starting materials.

**Fig. 8:**
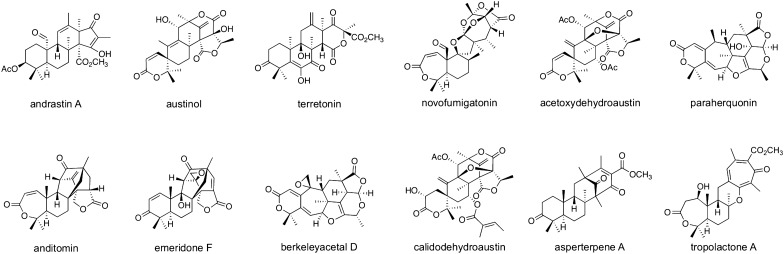
Representative DMOA-derived meroterpenoids

**Fig. 9:**
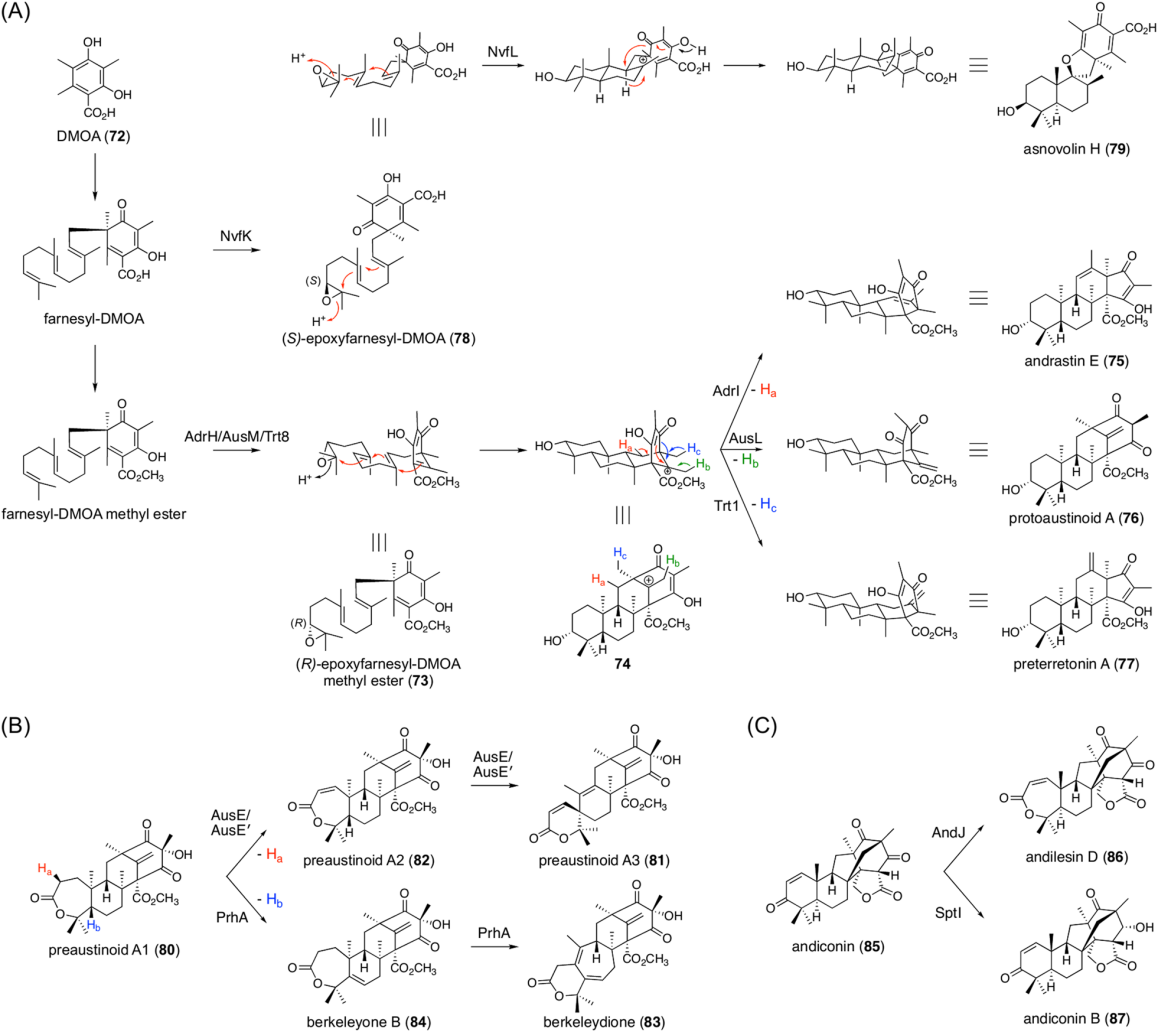
Pathway branching in the biosynthesis of DMOA-derived meroterpenoids. **A** Reactions catalyzed by terpene cyclases. **B** Reactions catalyzed by AusE/AusE′ and PrhA. **C** Reactions catalyzed by AndJ and SptI

One of the most intensively studied classes of fungal meroterpenoids comprises those derived from the aromatic polyketide 3,5-dimethylorsellinic acid (**72**; DMOA), and many DMOA-derived meroterpenoids possess a unique and complicated molecular skeleton (Fig. 8). A key factor generating the structural diversity of DMOA-derived metabolites is the existence of terpene cyclases with different activities. In the biosynthesis of andrastin A, austinol, and terretonin, (*R*)-epoxyfarnesyl-DMOA methyl ester (**73**) serves as a common intermediate but is cyclized into different products, generating the biosynthesis branching point (Fig. 9A) [74, 75]. Three terpene cyclases, AdrI, AusL, and Trt1, all accept **73** as a substrate and cyclize it into the tetracyclic carbocationic species **74**; however, in the final step of the reaction, these enzymes perform deprotonation at different positions to yield andrastin E (**75**), protoaustinoid A (**76**), and preterretonin A (**77**), respectively. Intriguingly, the terpene cyclase involved in novofumigatonin biosynthesis utilizes (*S*)-epoxyfarnesyl-DMOA (**78**) with a free carboxy group as a substrate to form asnovolin H (**79**) [76], although methyl-esterification is essential for the activities of AdrI, AusL, and Trt1 [74, 75]. Additionally, based on the cyclized product structures, AdrI, AusL, and Trt1 accept (*R*)-epoxide, whereas (*S*)-epoxide is used by NvfI, indicating the presence of epoxidases with different stereoselectivities. In a recent study by Mitsuhashi *et. al.*, synthetic epoxyfarnesyl-DMOA methyl ester molecules, including unnatural isomers, were reacted with a series of meroterpenoid cyclases, resulting in the production of several new meroterpenoid species generated by new cyclization modes [77]. Thus, enzymes that catalyze cyclization reactions in an unprecedented manner could be further discovered from unelucidated DMOA-derived meroterpenoid pathways.

Pathway branching in DMOA-derived meroterpenoid pathways also occurs at a later biosynthesis stage. Preaustinoid A1 (**80**) is a common biosynthetic precursor of several meroterpenoids (Fig. 9B), such as austinol, paraherquonin, and berkeleyacetals. The αKG-dependent dioxygenases AusE and AusE′, which are involved in the biosynthesis of austinol and acetoxydehydroaustin, respectively, accept **80** to catalyze two successive oxidations to afford preaustinoid A3 (**81**) with the characteristic spirolactone system via preaustinoid A2 (**82**) [78, 79]. Meanwhile, PrhA, a homologue of AusE/AusE′, utilizes the same substrate but performs a distinct oxidative rearrangement to yield berkeleydione (**83**) harboring the cycloheptadiene core via berkeleyone B (**84**) [79]. Intriguingly, AusE′ and PrhA share > 90% sequence identity, and a structural biology study revealed that only three amino acid residues in the enzyme active site are crucial for product selectivity [80]. Andiconin (**85**) serves as a common biosynthetic intermediate of anditomin and emeridone F (Fig. 9C) [81, 82]. In the anditomin pathway, AndJ, which acts as a Baeyer–Villiger monooxygenase, transforms **85** into andilesin D (**86**) with a seven-membered lactone ring [81]. Emeridone biosynthesis lacks an AndJ homologue, and **85** is accepted by the SDR SptI to be converted to andiconin B (**87**) [82]. It should be noted that the SptI homologue in the anditomin pathway, AndI, does not accept **85** as a substrate but acts only after the AndJ-catalyzed reaction.

**Fig. 10:**
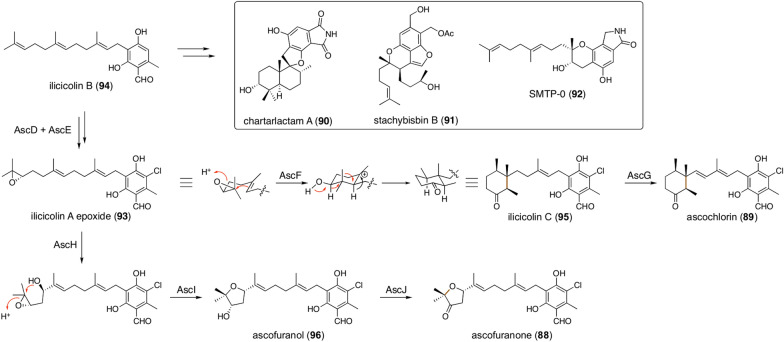
Biosynthesis of ilicicolin B-derived meroterpenoids

Polyketides other than DMOA can also be employed as the non-terpenoid portion of fungal meroterpenoids. Orsellinic acid serves as the precursor of diverse fungal meroterpenoids such as ascofuranone (**88**), ascochlorin (**89**), chartarlactam A (**90**), stachybisbin B (**91**), and SMTP-0 (**92**) (Fig. 10). Among these metabolites, the complete biosynthetic pathways of **88** and **89** have recently been elucidated [83], exemplifying pathway branching occurring in a single fungus. In the biosynthesis of these two meroterpenoids, orsellinic acid is converted to ilicicolin A epoxide (**93**) as a common precursor via ilicicolin B (**94**), which is also a biosynthetic intermediate of many other orsellinate-derived fungal meroterpenoids [84]. In the ascochlorin pathway, the terpene cyclase AscF cyclizes the farnesyl moiety of **93** to provide ilicicolin C (**95**) with a cyclohexanone ring, which subsequently undergoes dehydrogenation catalyzed by the P450 AscG to yield **89**. Ascofuranone biosynthesis involves three dedicated enzymes encoded by a separate gene cluster from that of **89**. In this process, **93** is first hydroxylated by the P450 AscH, followed by heterocyclization catalyzed by AscI to generate ascofuranol (**96**). Interestingly, AscI does not display sequence similarity with AscF and the terpene cyclases involved in the DMOA-derived metabolite pathways but is homologous to CtvD and AurD, the epoxide hydrolases involved in the biosynthesis of citreoviridin and aurovertin, respectively [85, 86]. Finally, the SDR AscJ performs an alcohol dehydrogenation reaction to complete the biosynthesis.

**Fig. 11:**
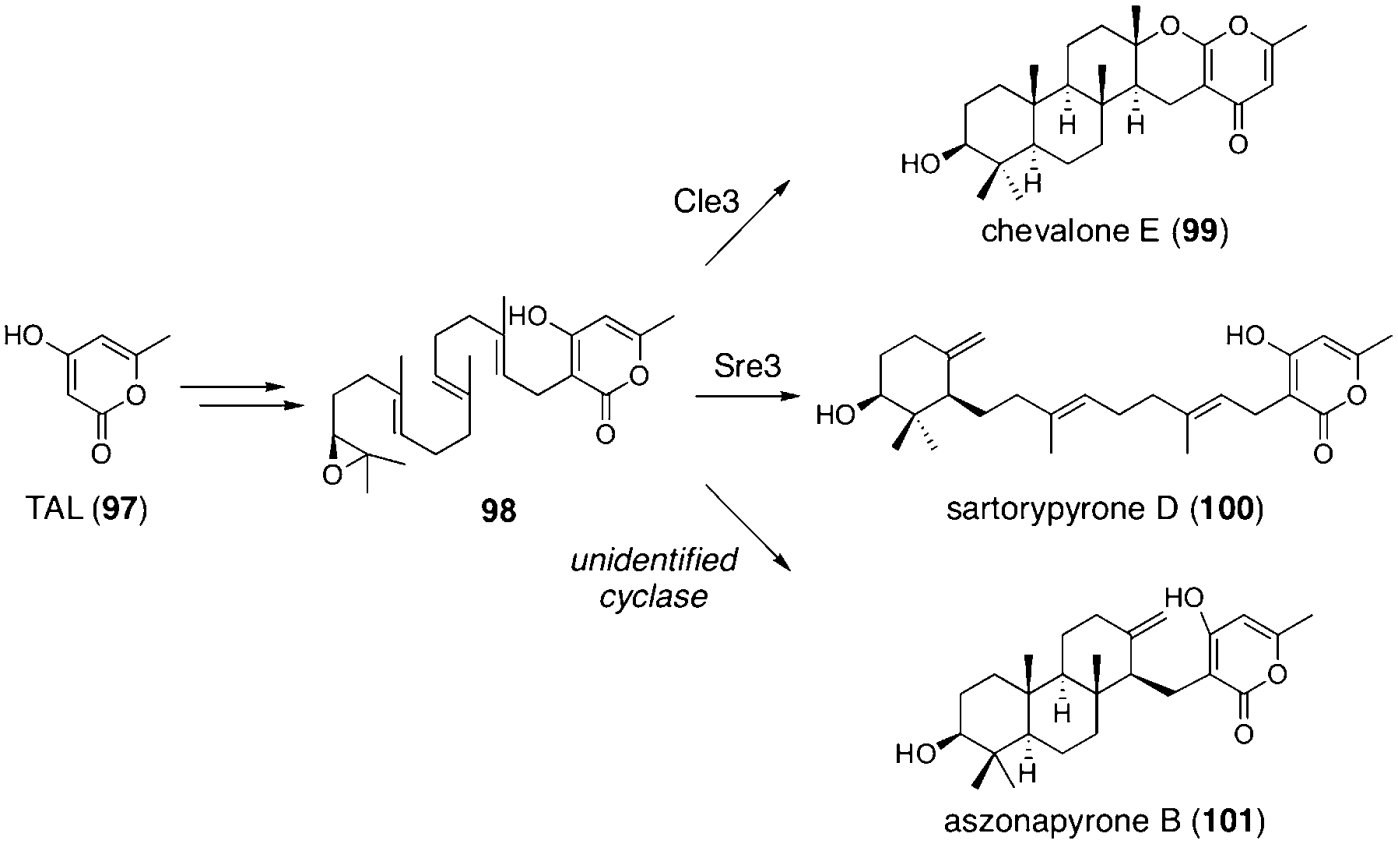
Terpene cyclases involved in the biosynthesis of TAL-derived meroterpenoids

Triacetic acid lactone (**97**; TAL) is another polyketide used as a fungal meroterpenoid building block. In the biosynthetic processes of known TAL-derived meroterpenoids, **97** undergoes geranylgeranylation and epoxidation to provide a substrate for terpene cyclases, **98** (Fig. 11) [87]. Chevalone biosynthesis adopts the terpene cyclase Cle3 to yield the pentacyclic product chevalone E (**99**), whereas Sre3, which is responsible for the sartorypyrone pathway, synthesizes sartorypyrone D (**100**), which possesses the monocyclic terpenoid moiety. The terpene cyclase for aszonapyrone biosynthesis appears to have another function in producing the tetracyclic aszonapyrone B (**101**) [88]; however, this function has yet to be identified.

**Fig. 12:**
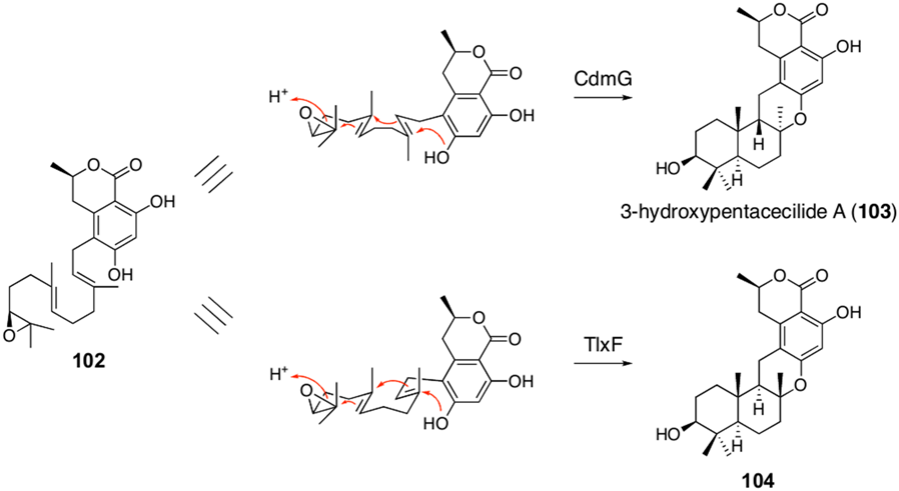
Terpene cyclases involved in the biosynthesis of chrodrimanins and talaromyolides

Another example in which terpene cyclases are responsible for pathway divergence can be found in the biosynthesis of chrodrimanins and talaromyolides (Fig. 12) [89, 90]. Both biosynthetic pathways adopt the identical precyclized intermediate **102** derived from the polyketide 6-hydroxymellein; however, terpene cyclization occurs in different ways. In chrodrimanin biosynthesis, the terpene cyclase CdmG folds the farnesyl moiety in the *chair-boat* conformation to yield 3-hydroxypentacecilide A (**103**), whereas TlxF involved in the talaromyolides pathway performs *chair-chair* cyclization to give **104**.

**Fig. 13:**
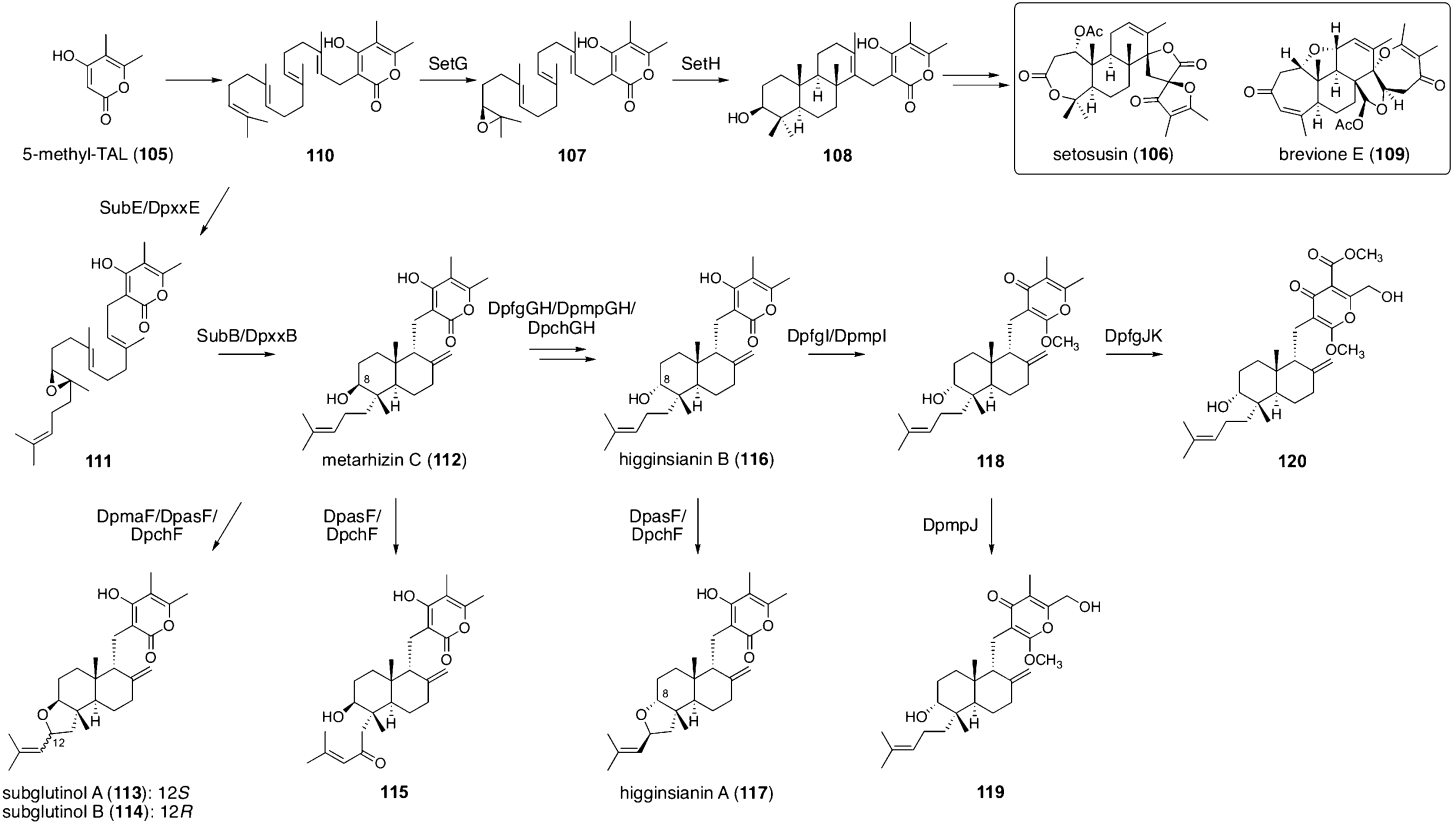
Biosynthesis of 5-methyl TAL-derived meroterpenoids. Note that xx in DpxxB/DpxxE should be substituted with one of the following: as, ch, fg, ma, and mp

A methylated analogue of TAL (**97**), 5-methyl TAL (**105**), is often found in fungal meroterpenoid structures, and in recent years, the complete biosynthetic pathways of several 5-methyl TAL-derived meroterpenoids have been revealed (Fig. 13) [91–93]. In the biosynthesis of setosusin (**106**), **105** undergoes a series of reactions similar to those in TAL-derived meroterpenoid pathways to provide a precyclized intermediate **107**, which is accepted by the terpene cyclase SetH to give **108** with a tricyclic terpenoid portion [93]. This cyclized product is also used as the precursor for other fungal metabolites, such as brevione E (**109**) [94], although brevione E biosynthetic genes have not been reported. The biosynthesis of other 5-methyl TAL-derived meroterpenoids, such as subglutinols, higginsianins, and metarhizins, branches from the setosusin/brevione pathways at an early biosynthesis stage. In the biosynthetic processes of these molecules, epoxidation occurs at the second double bond from the geranylgeranyl chain terminus of **110** to afford **111**, which is cyclized into metarhizin C (**112**) with a bicyclic terpenoid moiety. In a recent study by Tsukada *et. al*. [92], biosynthetic genes for metarhizin C-derived metabolites from five fungal species were examined and heterologously expressed, leading to the production of several known and new meroterpenoids. The FAD-dependent oxidoreductase DpxxF (xx = ma, as, or ch) conducts oxidative cyclization to synthesize subglutinols A (**113**) and B (**114**) from **112**. DpasF or DpchF can also install a carbonyl group onto the dimethylallyl portion of **112** to yield a previously undescribed meroterpenoid **115**. The SDRs DpxxG and DpxxH (xx = fg, mp, or ch) invert the stereochemistry of **112** at the C-8 position to give higginsianin B (**116**). Compound **116** is oxidized by DpasF or DpchF to provide higginsianin A (**117**), which is the C-8 epimer of **113**. Compound **116** also serves as the precursor for three new meroterpenoids **118**–**120**, which are synthesized by catalysis of the methyltransferase DpfgI or DpmpI, the P450 DpfgJ or DpmpJ, and the methyltransferase DpfgK.

**Fig. 14:**
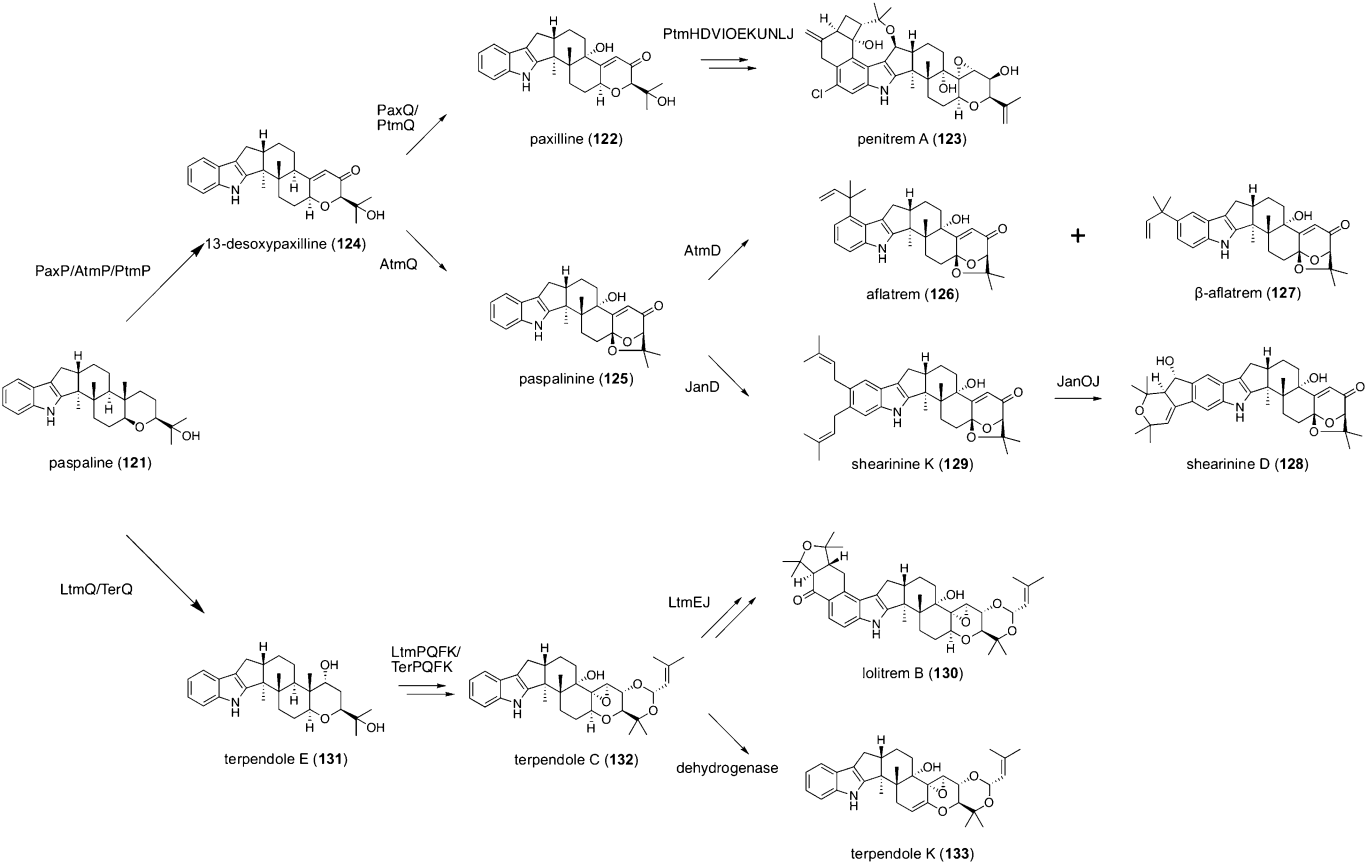
Biosynthesis of paspaline-derived indole diterpenoids

Although the majority of fungal meroterpenoids originate from polyketide and terpenoid pathways, non-polyketide molecules can also be observed in meroterpenoid structures as non-terpenoid moieties. Indole diterpenoids are a large group of fungal meroterpenoids lacking a polyketide portion; instead, they possess an indole ring as a non-terpenoid moiety [95]. As observed in the biosynthesis of polyketide-derived meroterpenoids, the terpenoid chain cyclization mode contributes to the structural diversity of indole diterpenoids [96]. However, the majority of indole diterpenoids are biosynthesized via the hexacyclic molecule paspaline (**121**) (Fig. 14), which is generated from indole-3-glycerol phosphate by four dedicated enzymes [97, 98]. In an indole diterpenoid biosynthesis branch, **121** is transformed into paxilline (**122**) by the catalysis of two P450s, PaxP and PaxQ [97]. Compound **122** further undergoes highly complicated tailoring reactions to provide penitrem A (**123**) [99]. The PaxP product 13-desoxypaxilline (**124**) also serves as a precursor of other indole diterpenoids, such as aflatrems and shearinines. In aflatrem biosynthesis, **124** is oxidized by the P450 AtmQ to afford paspalinine (**125**), which is further converted to aflatrem (**126**) and its isomer β-aflatrem (**127**) by the dimethylallyltryptophan synthase (DMATS)-type prenyltransferase AtmD [100–102]. JanD, an AtmD homologue involved in the biosynthesis of shearinine D (**128**), also accepts **125** as a substrate but performs diprenylatation to give shearinine K (**129**) [103], which undergoes oxidative tailoring reactions by the FMO JanO and the P450 JanJ to complete biosynthesis [104]. Meanwhile, the biosynthesis of lolitrem B (**130**) branches from the other pathways mentioned above just after paspaline formation. The P450 LtmQ, which is homologous to PaxQ and AtmQ, performs hydroxylation to provide terpendole E (**131**) [105, 106]. Compound **131** is subsequently transformed into terpendole C (**132**) by four enzymes, and finally, **130** is synthesized by the prenyltransferase LtmE and the P450 LtmJ from **132** [107, 108]. In *Chaunopycnis alba*, an uncharacterized dehydrogenase oxidizes **132** to yield terpendole K (**133**) [106].

## Converging pathways

Heterodimeric compounds, which are formed by the fusion of two metabolites derived from two distinct pathways, are often obtained during natural product isolation. Most heterodimeric natural products are often spontaneously formed due to reactive structural elements in the monomeric natural products that compose the heterodimer. However, in some biosynthetic processes, two or more compounds synthesized via separate pathways undergo an enzyme-catalyzed fusion reaction, expanding the structural diversity of natural products. This section summarizes representative converging pathways observed in fungal natural product biosynthesis and the enzymes responsible for the hybridization of two distinct natural products.

### Addition of a polyketide chain

**Fig. 15:**
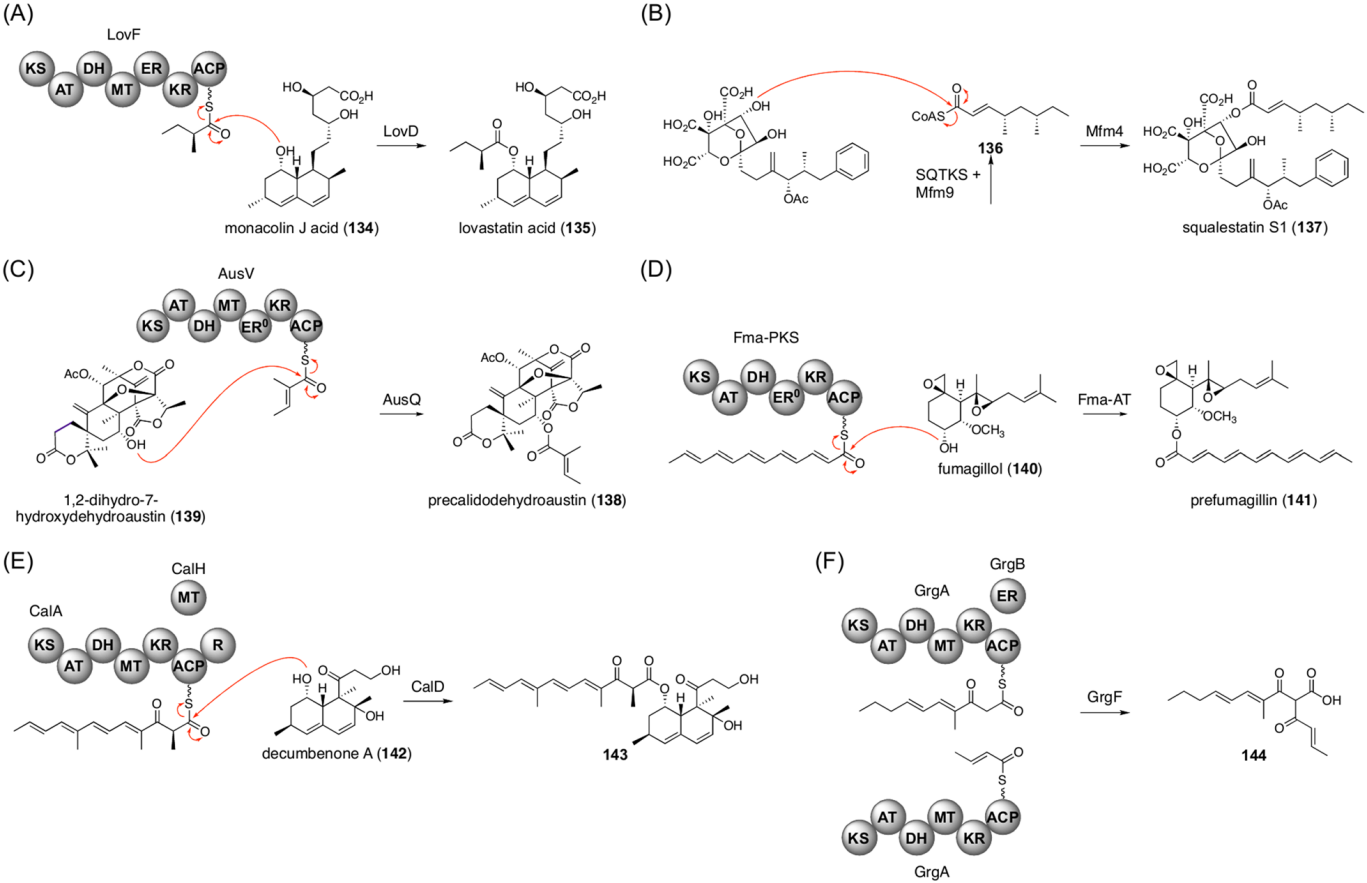
Additions of a polyketide chain during fungal natural product biosynthesis. Reactions catalyzed by **A** LovD, **B** Mfm4, **C** AusQ, **D** Fma-AT, **E** CalD, and **F** GrgF

One representative converging pathway in fungal natural product biosynthesis is the addition of a polyketide chain to a molecule synthesized in a separate biosynthetic route. One example is the biosynthesis of harzianum A (**54**), as mentioned above (Fig. 5C) [58], and similar reactions to attach a polyketide chain are found in many fungal natural product pathways (Fig. 15). In the last step of lovastatin biosynthesis, the acyltransferase LovD transfers the polyketide chain synthesized by the PKS LovF to introduce a methylbutanoyl moiety to monacolin J acid (**134**) to yield lovastatin acid (**135**) (Fig. 15A) [109]. Squalestatin biosynthesis involves the acyltransferase Mfm4 that utilizes the tetraketide CoA **136** as a substrate to yield squalestatin S1 (**137**); **136** is synthesized by the PKS squalestatin tetraketide synthase (SQTKS) and the CoA ligase Mfm9 (Fig. 15B) [110, 111]. In fungal meroterpenoid calidodehydroaustin biosynthesis (Fig. 8), the acyltransferase AusQ performs a transacylation reaction by utilizing the polyketide chain generated by the PKS AusV to give precalidodehydroaustin (**138**) from 1,2-dihydro-7-hydroxydehydroaustin (**139**) (Fig. 15C) [112]. AusQ is also known to catalyze acetylation reactions at the same position. The biosynthesis of another fungal meroterpenoid, fumagillin, adopts the acyltransferase Fma-AT to install the dodecapentaenoyl moiety, which is synthesized by the PKS Fma-PKS, onto fumagillol (**140**) to produce prefumagillin (**141**) (Fig. 15D) [113].

Calbistrins are structurally similar to lovastatin in that they have a polyketide chain attached to a decalin core; however, the biosynthesis of calbistrins and lovastatin somewhat differs. CalD, the enzyme responsible for the acylation reaction in the calbistrin pathway, performs a similar reaction to LovD to introduce the hexaketide moiety to decumbenone A (**142**) to yield **143** (Fig. 15E), although CalD does not exhibit sequence similarity with LovD [114]. Interestingly, the calbistrin biosynthetic gene cluster encodes a LovD homologue, CalJ, which is unable to perform acylation reactions but instead catalyzes the hydrolysis of the ester bond formed by the CalD-catalyzed reaction. Another intriguing feature of calbistrin biosynthesis is that both the decalin and polyene portions are generated from the single PKS CalA. The decalin portion is synthesized in a CalA and *trans*-acting enoylreductase (*trans*-ER) CalK collaboration, whereas the polyene hexaketide is formed by CalA and the *trans*-acting methyltransferase CalH. Thus, the PKS CalA contributes both to pathway branching and convergence in the biogenesis of calbistrins.

Gregatin biosynthesis provides a similar example in which a single PKS is responsible for the synthesis of two distinct polyketide chains that are eventually fused together (Fig. 15F) [115]. GrgA, the PKS involved in the gregatin pathway, synthesizes the longer pentaketide chain in the presence of the *trans*-ER GrgB, whereas it produces a shorter diketide chain when GrgB is absent. These two polyketide chains are fused by the α/β hydrolase fold enzyme GrgF to generate **144**.

### Incorporation of non-proteinogenic amino acids into NRPS assembly lines

One key feature of nonribosomal peptide biosynthesis, distinct from that of ribosomal synthesis, is that non-proteinogenic amino acids and non-amino acid molecules can be directly used as building blocks for peptide chain synthesis [116]. Non-standard amino acids found in nonribosomal peptides are often synthesized by dedicated enzymes prior to acceptance by the NRPS assembly line, thus exemplifying converging pathways in natural product biosynthesis.

**Fig. 16:**
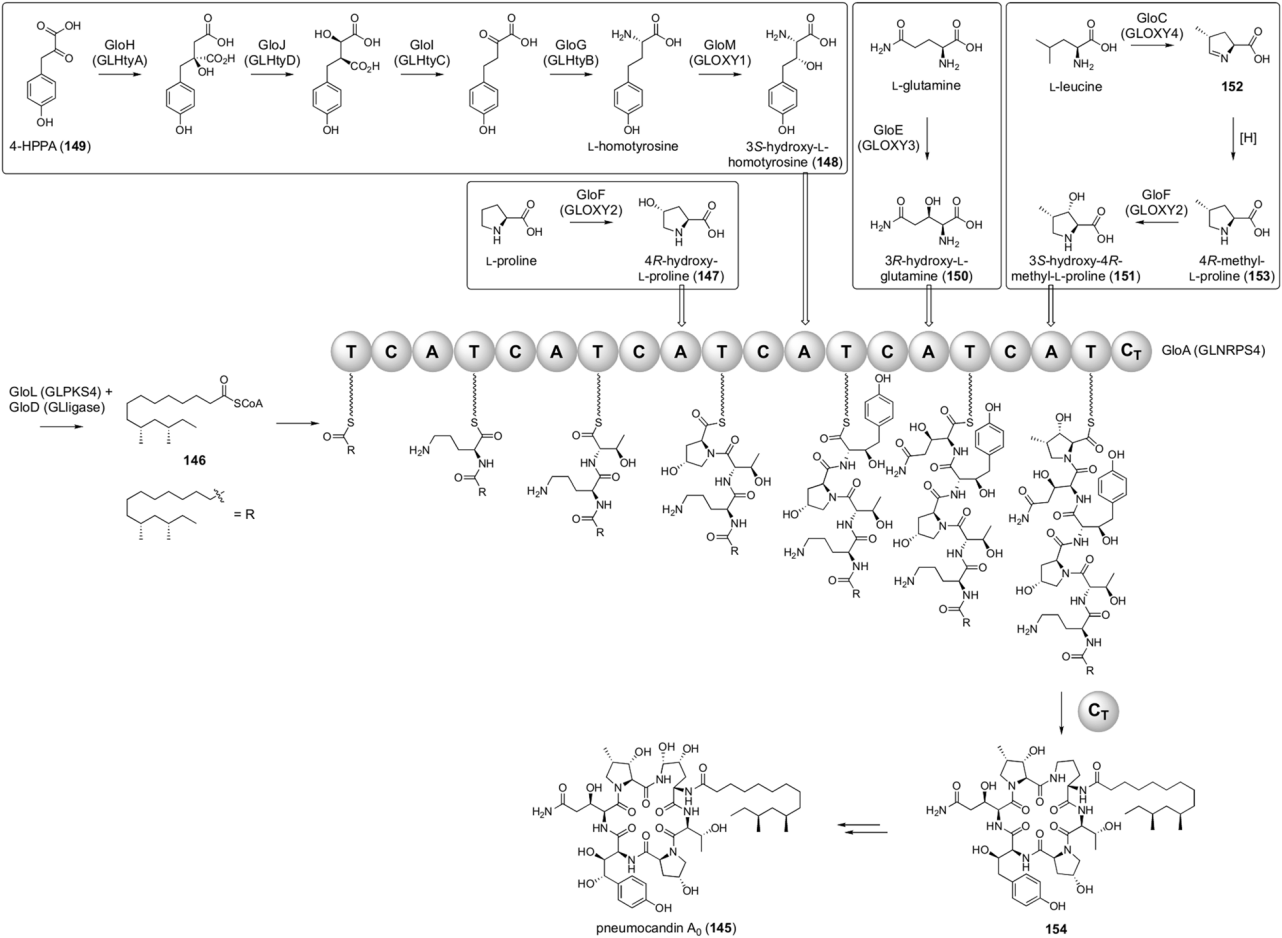
Biosynthesis of pneumocandin A_0_ (**145**)

Pneumocandin A_0_ biosynthesis is one of the most complicated examples in which the dedicated syntheses of unusual amino acids are required (Fig. 16). Pneumocandin A_0_ (**145**) is an antifungal agent in the echinocandin class, obtained from the fungus *Glarea lozoyensis* [117]. Pneumocandin A_0_ biosynthesis is initiated with the synthesis of dimethylmyristoyl-CoA (**146**) by the PKS GloL (GLPKS4) and the CoA ligase GloD (GLligase), and **146** is loaded onto the NRPS GloA (GLNRPS4) as a starter unit for peptide chain synthesis [118, 119]. GloA consists of six modules, five of which accept a non-proteinogenic amino acid. Among these five non-proteinogenic amino acids, ornithine, accepted by the first A domain, is an important primary metabolite sufficiently present in cells. However, the remaining four amino acids need to be prepared by utilizing dedicated enzymes encoded by the pneumocandin biosynthetic gene cluster. The third A domain activates 4*R*-hydroxy-l-proline (**147**), which is synthesized by l-proline hydroxylation catalyzed by the αKG-dependent dioxygenase GloF (GLOXY2) [120]. The fourth A domain accepts 3*S*-hydroxy-l-homotyrosine (**148**), which is synthesized from 4-hydroxyphenylpyruvic acid (**149**; 4-HPPA) by five enzymes, GloH, -J, -I, -G, and -M (GLHtyA, -HtyD, -HtyC, -HtyB, and -OXY1) [119, 121, 122]. The αKG-dependent dioxygenase GloE (GLOXY3) hydroxylates l-glutamine to provide 3*R*-hydroxy-l-glutamine (**150**) [123], which is loaded onto the fifth module. The sixth A domain substrate, 3*S*-hydroxy-4*R*-methyl-l-proline (**151**), is generated from l-leucine. In this transformation, l-leucine is oxidized by the αKG-dependent dioxygenase GloC (GLOXY4), and the resultant cyclic imine **152** is reduced to 4*R*-methyl-l-proline (**153**) by an uncharacterized enzyme [119, 122]. GloF, which is also used in 4*R*-hydroxy-l-proline synthesis, hydroxylates **153** to yield **151** [120]. The linear peptide chain synthesized by GloA undergoes C_T_ domain-catalyzed cyclization to be released from the NRPS enzyme to provide the cyclic peptide **154**. The cyclic peptide undergoes further oxidative tailoring reactions to finally provide **145**. Similar biosynthetic strategies incorporating unusual amino acids or starter units are also found in other fungal NRPS pathways, such as the biosynthesis of echinocandins [121, 122] and leucinostatins [124].

**Fig. 17:**
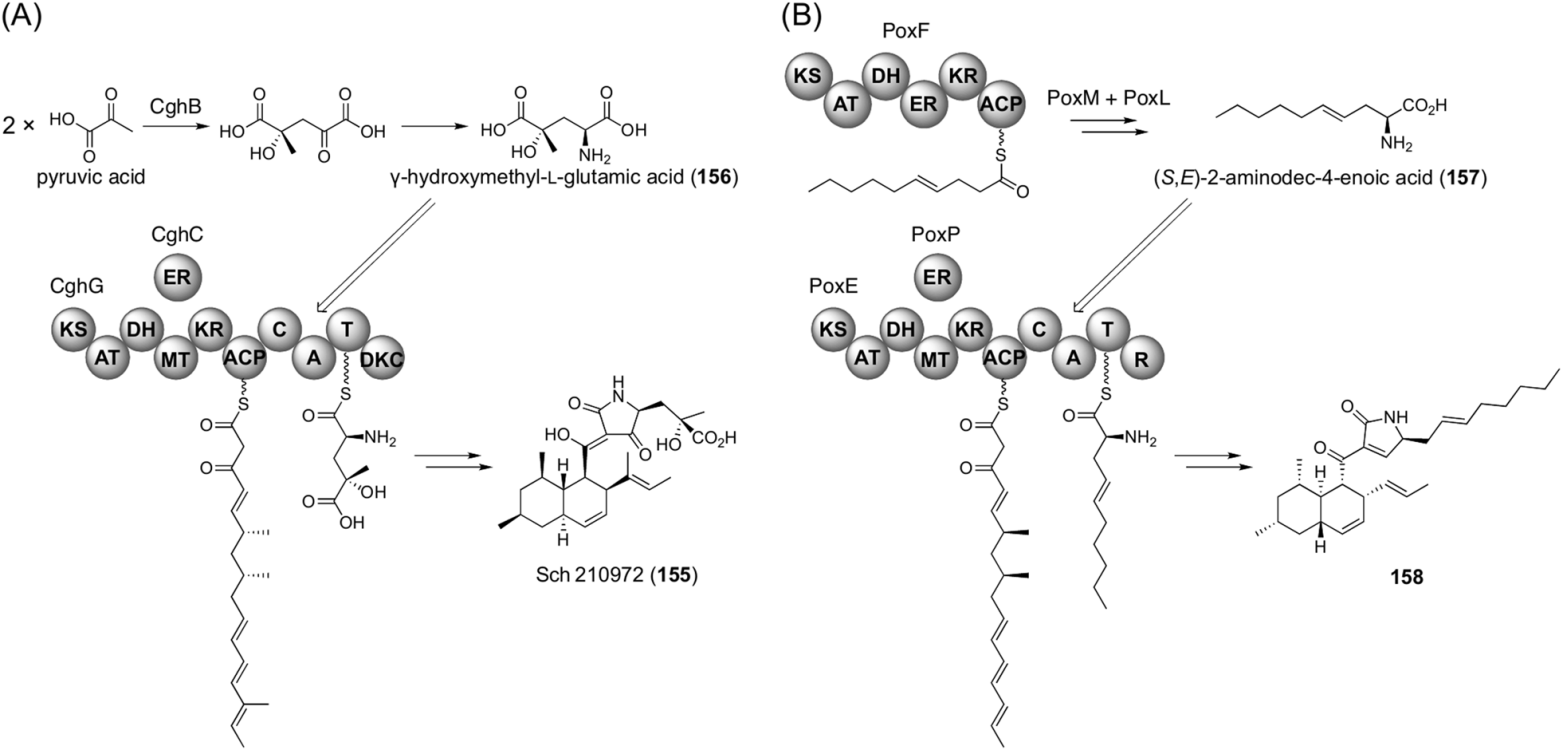
Biosynthesis of fungal polyketide-nonribosomal peptide hybrids with a non-proteinogenic amino acid moiety. **A** Biosynthesis of Sch 210972 (**151**). **B** Early-stage biosynthesis of oxaleimides

Some fungal PKS-NRPS hybrids also accept an unusual amino acid synthesized by dedicated enzymes encoded by a gene cluster containing the PKS-NRPS gene (Fig. 17). In the biosynthesis of Sch 210972 (**155**), γ-hydroxymethyl-l-glutamic acid (**156**) is accepted by the PKS-NRPS CghG A domain (Fig. 17A) [125]. The biosynthesis of this non-proteinogenic amino acid involves the condensation of two pyruvic acid molecules catalyzed by the aldolase CghB, followed by a stereoselective transamination reaction. It has been proposed that transamination is performed by a transaminase encoded outside the biosynthetic gene cluster. Another similar example is found in the biosynthesis of oxaleimides, in which the PKS-NRPS PoxE utilizes (*S*,*E*)-2-aminodec-4-enoic acid (**157**) as a substrate of the A domain to yield **158** (Fig. 17B) [126]. The amino acid backbone structure is generated by the PKS PoxF, which is modified by the P450 PoxM and the aminotransferase PoxL.

### Communesin B

**Fig. 18:**
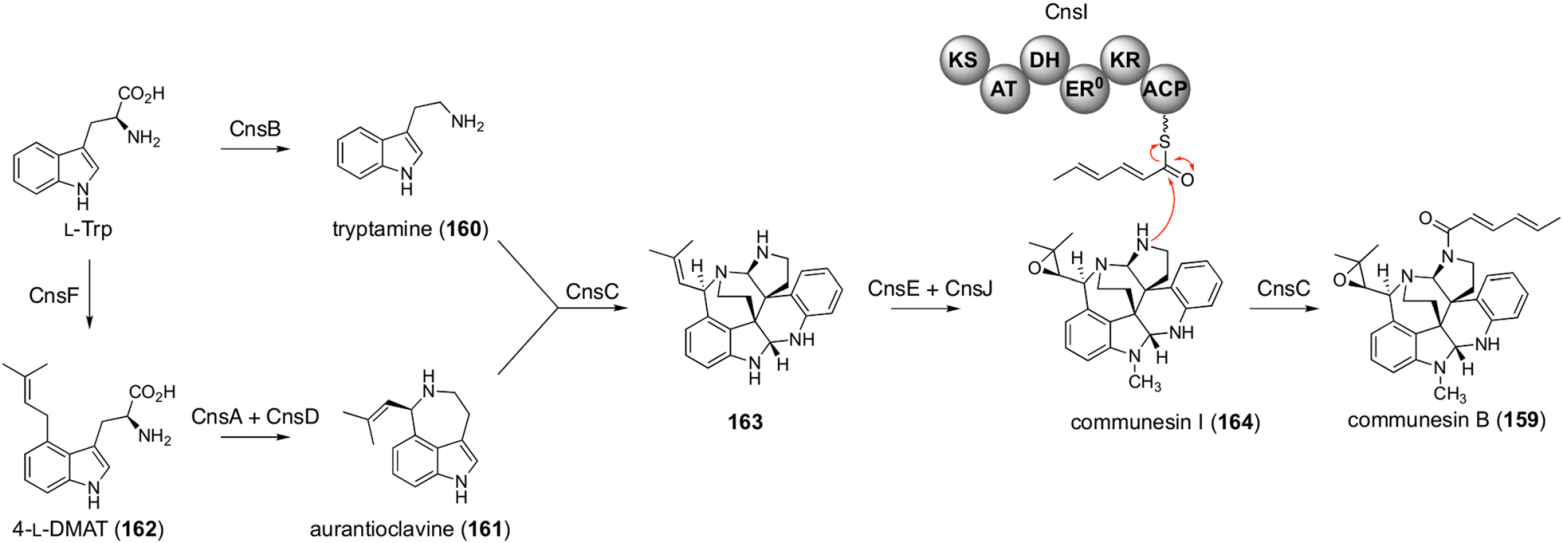
Biosynthesis of communesin B (**159**)

Communesins are dimeric indole alkaloids isolated from *Penicillium* fungi [127–130]. Some of them, such as communesin B (**159**), harbor the hexadienoyl moiety, which originates from the polyketide pathway. The communesin pathway involves both pathway branching and convergence to furnish the unique molecular architecture [131, 132]. The communesin core structure is derived from two l-tryptophan molecules, each undergoing different pathways to provide monomeric units of communesins (Fig. 18) [131]. In one branch, l-tryptophan is converted to tryptamine (**160**) by the tryptophan decarboxylase CnsB. l-Tryptophan is also transformed into aurantioclavine (**161**) via 4-dimethylallyl-l-tryptophan (**162**; 4-l-DMAT) by catalysis with the DMATS CnsF, the FMO CnsA, and the catalase CnsD. In the subsequent biosynthetic step, **160** and **161** undergo an oxidative coupling reaction catalyzed by the P450 CnsC to afford the communesin backbone structure **163** [132], which is further converted to communesin I (**164**) by the methyltransferase CnsE and the αKG-dependent dioxygenase CnsJ. Finally, the acyltransferase CnsC performs *N*-acylation by utilizing the hexadienoyl chain synthesized by the PKS CnsI to yield **159**.

### Flavunoidine

**Fig. 19:**
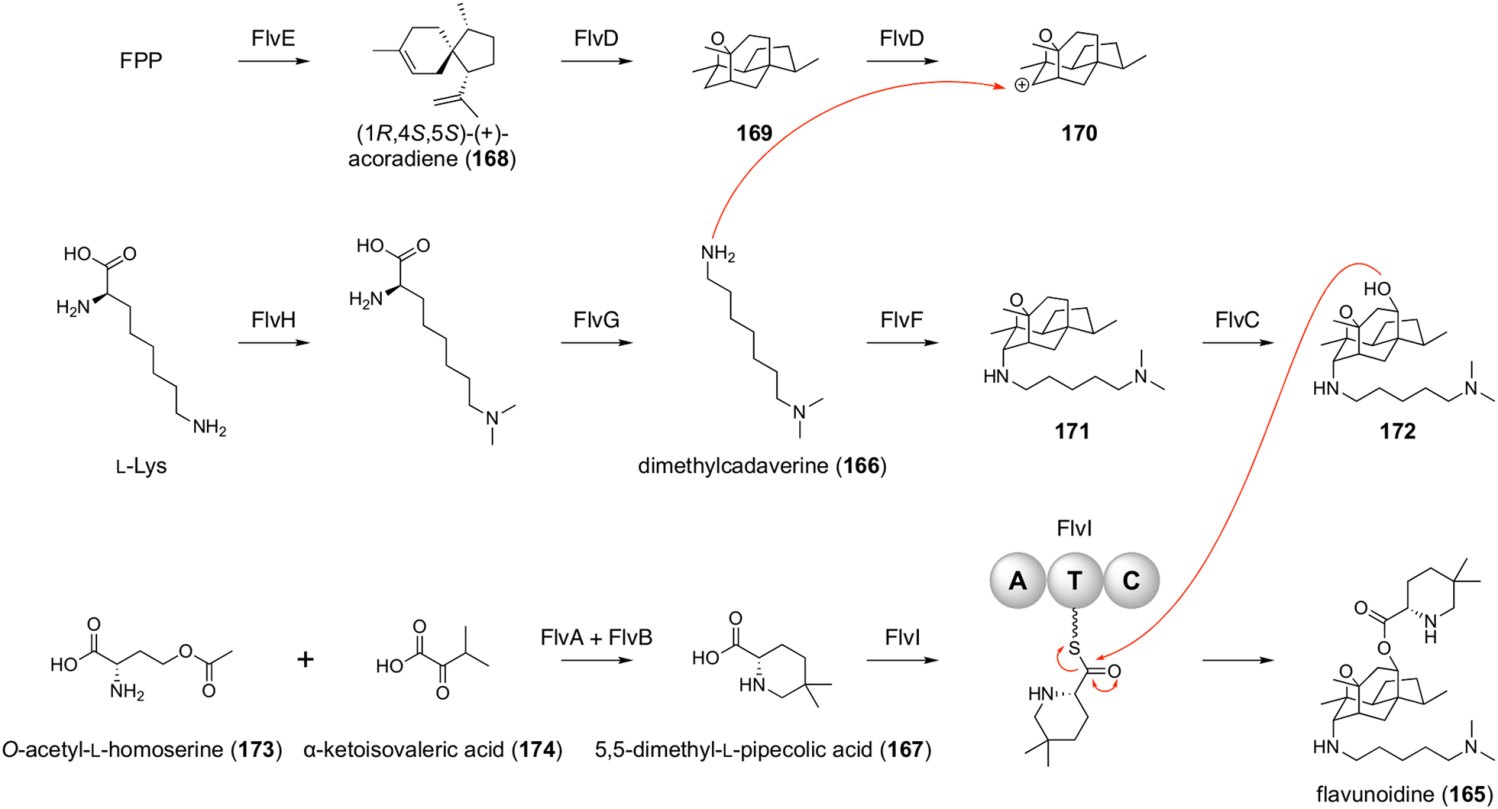
Biosynthesis of flavunoidine (**165**)

Flavunoidine (**165**) is obtained by genome mining in the fungus *Aspergillus flavus* and features a sesquiterpenoid core modified with dimethylcadaverine (**166**) and 5,5-dimethyl-l-pipecolic acid (**167**) [133]. It is a rare example of terpenoid-amino acid conjugated natural products. Compound **165** is generated by the fusion of three separately synthesized building blocks (Fig. 19). In one of the three pathways, l-lysine is converted into **166** by the *N*-methyltransferase FlvH and the decarboxylase FlvG. Meanwhile, the terpene cyclase FlvE cyclizes FPP into (1*R*,4*R*,5*S*)-( +)-acoradiene (**168**). The P450 FlvD accepts the sesquiterpene hydrocarbon **168** as a substrate to transform it into **169** with a tetracyclic cage. The sesquiterpenoid is further oxidized to the carbocationic species **170**, which then reacts with **166**; this nucleophilic addition is mediated by the terpene cyclase-like enzyme FlvF. This hybrid molecule **171** is hydroxylated by the P450 FlvC to generate **172**, which is further combined with **167** to yield the end product **165**. The unusual amino acid **167** is synthesized from *O*-acetyl-l-homoserine (**173**) and α-ketoisovaleric acid (**174**) by FlvA, the chimeric protein of PLP-dependent lyase and αKG-dependent dioxygenase, and the SDR FlvB. Compound **167** is then loaded onto the single-module NRPS FlvI, which performs the esterification reaction to afford **165**.

### Other converging pathways

**Fig. 20:**
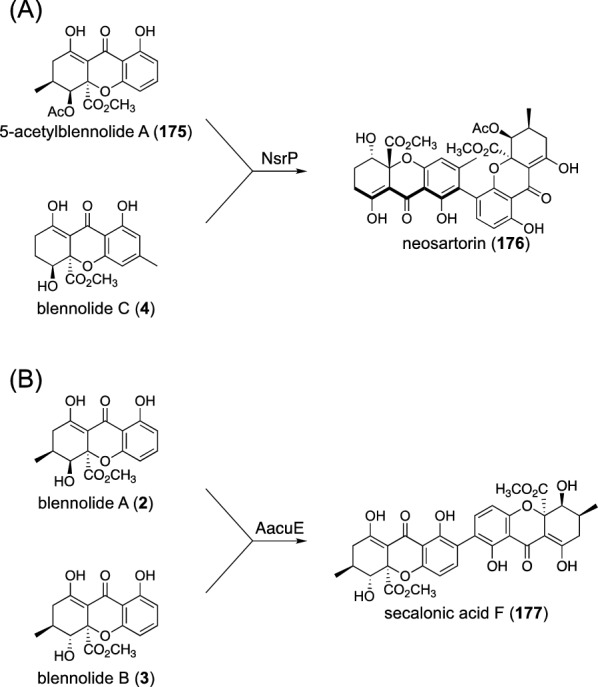
P450-catalyzed oxidative coupling reactions in fungal THX heterodimer biosynthesis. Reactions catalyzed by **A** NsrP and **B** AacuE

In addition to the biosynthetic pathways described above, fungi accomplish pathway convergence in diverse ways. Fungal THX dimer biosynthesis is one such example. As mentioned above, fungal THX biosynthesis occurs in a highly branching way (Fig. 3); however, these monomeric THXs often undergo (hetero)dimerization reactions to provide THX (hetero)dimers, and the biosynthesis of THX heterodimers can be regarded as converging pathways. The P450 NsrP performs oxidative coupling of 5-acetylblennolide A (**175**) and blennolide C (**4**) to complete neosartorin (**176**) biosynthesis (Fig. 20A) [12]. Similarly, AacuE, an NsrP homologue responsible for secalonic acid biosynthesis, can accept blennolides A (**2**) and B (**3**) as substrates to generate secalonic acid F (**177**) (Fig. 20B) [16]. It should be noted that these P450s appear to utilize several different substrates to perform both homo- and heterodimerization reactions. For example, AacuE can synthesize secalonic acid D [16], a homodimer of **3** and a major product of the *A. aculeatus* secalonic acid pathway [134].

**Fig. 21:**
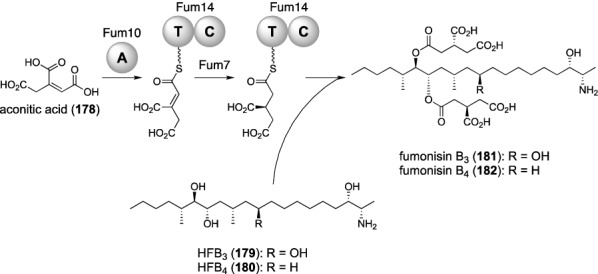
Biosynthesis of fumonisins

Fumonisins are mycotoxins mainly derived from *Fusarium* species and possess characteristic tricarballylic ester moieties [135]. The tricaballylate portion is derived from aconitic acid (**178**), which is activated by the standalone A domain Fum10 and subsequently loaded onto the NRPS-like enzyme Fum14 with T-C domain organization (Fig. 21) [136]. It has been proposed that the tricaballylate bound to Fum14 undergoes enoylreduction catalyzed by the iron-containing dehydrogenase/reductase Fum7 [137]. The Fum14 C domain would then utilize hydrolyzed fumonisin B_3_ (**179**; HFB_3_) or hydrolyzed fumonisin B_4_ (**180**; HFB_4_), which are synthesized in a separate pathway, to perform the esterification reaction. The resultant monoacylated products would again be accepted by the C domain to provide fumonisin B_3_ (**181**) or fumonisin B_4_ (**182**).

**Fig. 22:**
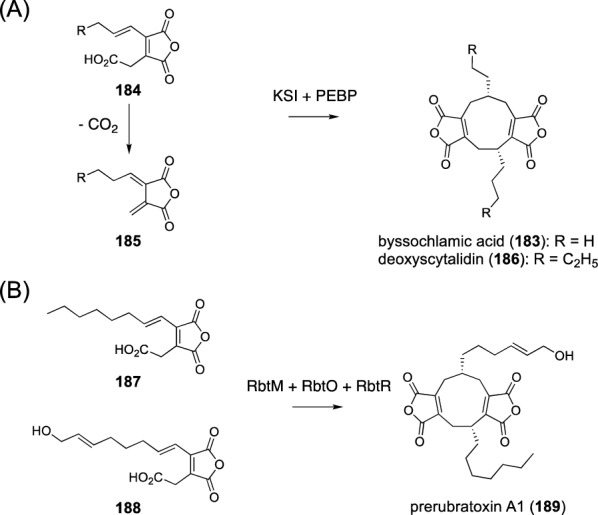
Biosynthesis of maleidrides. **A** Biosynthesis of byssochlamic acid (**183**) and deoxyscytalidin (**186**). **B** Biosynthesis of prerubratoxin A1 (**189**)

Maleidrides are carbocyclic molecules harboring one or two maleic anhydride groups [138], and their biosynthesis involves an enzyme-catalyzed heterodimerization reaction to synthesize the core structure. In the biosynthesis of byssochlamic acid (**183**), butenyl carboxymethyl maleic anhydride (**184**) and its decarboxylated form **185** are enzymatically hybridized (Fig. 22A) [139]; the maleic anhydride derivative **184** originates from hexenoate synthesized by a PKS and oxaloacetate. The reaction to generate **183** is catalyzed by the ketosteroid isomerase (KSI)-like proteins BfL6 and BfL10, and the product yield is significantly increased in the presence of phosphatidylethanolamine-binding proteins (PEBPs) BfL5 and BfL9. Deoxyscytalidin (**186**) biosynthesis occurs in a similar manner to **183**, except that octanoate is used as a building block instead of hexenoate (Fig. 22A) [140]. The fusion reaction in the scytalidin pathway is performed by the KSI ScyR6 and the PEBPs ScyL1 and ScyR12. Rubratoxin A biosynthesis constructs the maleidride scaffold in a similar manner with the aid of the KSI RbtR and the PEBPs RbtM and RbtO by utilizing two carboxymethyl maleic anhydride molecules with slightly different side chains, **187** and **188**, to afford prerubratoxin A1 (**189**) (Fig. 22B) [141].

**Fig. 23:**
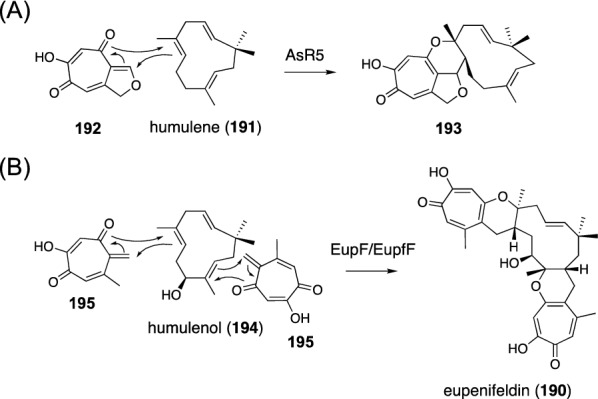
Hetero [4 + 2] cycloaddition reactions in fungal meroterpenoid biosynthesis. Reactions catalyzed by **A** AsR5 and **B** EupF/EupfF

Xenovulene A and eupenifeldin (**190**) are polyketide-derived fungal meroterpenoids synthesized in a distinct manner from those described above. The biosynthesis of these molecules employs a class I terpene cyclase, and therefore, the resultant terpene products need to be hybridized with a polyketide molecule at a later biosynthesis stage. In the biosynthesis of xenovulene A, the sesquiterpene hydrocarbon humulene (**191**) and the tropolone quinomethide (**192**) undergo a hetero [4 + 2] cycloaddition reaction catalyzed by AsR5 to give **193** with the meroterpenoid scaffold (Fig. 23A) [142]. The eupenifeldin pathway also adopts a homologous enzyme EupF or EupfF, which catalyzes hetero [4 + 2] cycloaddition reactions at two distinct positions of the sesquiterpene alcohol humulenol (**194**) using two tropolone orthoquinone molecules (**195**) (Fig. 23B) [143, 144].

**Fig. 24:**
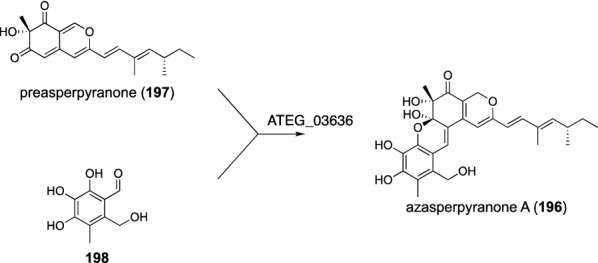
Biosynthesis of azasperpyranone A (**196**)

Azasperpyranone A (**196**) is an azaphilone family natural product biosynthesized by the fusion of preasperpyranone (**197**) and a polyketide-derived aromatic aldehyde **198**, which, unlike the other cases covered in this review, are generated by two separate gene clusters (Fig. 24) [145]. However, a gene deletion experiment indicated that ATEG_03636 is responsible for this heterodimerization reaction. ATEG_03636 does not exhibit sequence similarity to any characterized proteins, and the detailed mechanism by which the enzyme performs the hybridization reaction has yet to be elucidated.

## Concluding remarks

In this review, we summarize several representative examples of branching and converging pathways found in fungal natural product biosynthesis. Core synth(et)ases, such as PKSs, NRPSs, and terpene cyclases, undoubtfully contribute to the structural diversity of natural products by creating a large number of branching pathways, which is achieved by the diverse programming rules adopted by core synth(et)ases. However, pathway branching and converging at mid- and late-stage biosynthesis, which are performed by a variety of tailoring enzymes, are other major factors that generate the natural product diversity and complexity. It should be noted that only slight genetic changes can sometimes cause a pathway divergence; for example, the divergence of paraherquonin and acetoxydehydroaustin pathways in *Penicillium brasilianum* stems from the change of a few amino acid residues in the key dioxygenases [79]. The presence of diverse branching and converging pathways might be attributed to nature’s evolutionary efforts to synthesize diverse molecules from a relatively small number of available starting materials. Given the recent rapid accumulation and advances in genome editing and heterologous expression technologies [146, 147], an increasing number of branching and converging biosynthesis pathways will be identified and characterized in the near future, which could lead to the discovery of novel biosynthetic mechanisms for pathway branching or convergence. The elucidation and understanding of such biosynthetic pathways will also facilitate the construction of artificial branching and converging metabolic pathways, as exemplified by the expansion of meroditerpenoid pyrone pathways [92], the evolution of the acyltransferase LovD into a simvastatin synthase [148], and the side-chain engineering of pneumocandins [149]. Future studies might discover more branching and converging pathways and rationally engineer biosynthesis to further expand natural product diversity and provide useful molecules in an efficient and selective manner.

## Abbreviations

4-HPPA: 4-Hydroxyphenylpyruvic acid
αKG: α-Ketoglutarate
A: Adenylation
C: Condensation
CoA: Coenzyme A
C_T_: Terminal condensation-like
DMAPP: Dimethylallyl pyrophosphate
DMAT: Dimethylallyltryptophan
DMATS: Dimethylallyltryptophan synthase
DMOA: 3,5-Dimethylorsellinic acid
DON: Deoxynivalenol
EPT: 12,13-Epoxytrichothec-9-ene
ER: Enoylreductase
FMO: Flavin-dependent monooxygenase
FPP: Farnesyl pyrophosphate
KSI: Ketosteroid isomerase
NIV: Nivalenol
NRPS: Nonribosomal peptide synthetase
PEBP: Phosphatidylethanolamine-binding proteins
PKS: Polyketide synthase
R: Reductase
SDR: Short-chain dehydrogenase/reductase
SQTKS: Squalestatin tetraketide synthase
T: Thiolation
TAL: Triacetic acid lactone
THX: Tetrahydroxanthone
